# Smart responsive biomaterials for spatiotemporal modulation of functional tissue repair

**DOI:** 10.1016/j.mtbio.2025.102063

**Published:** 2025-07-09

**Authors:** Xu He, Zeyu Han, Yunxuan Ruan, Zijie Wang, Bo Liao, Xinhe Li, Jindong Tan, Xiaoyu Han, Jieliang Shen, Dingqun Bai

**Affiliations:** a Department of Rehabilitation Medicine, Key Laboratory of Physical Medicine and Precision Rehabilitation of Chongqing Municipal Health Commission, The First Affiliated Hospital of Chongqing Medical University, No.1 Youyi Road, Yuzhong District, Chongqing, 400016, PR China; b Department of Rehabilitation Medicine, Bishan Hospital of Chongqing Medical University, Bishan Hospital of Chongqing, Chongqing, 402760, PR China; c Department of Orthopaedics, Shanghai Key Laboratory for Prevention and Treatment of Bone and Joint Diseases, Shanghai Institute of Traumatology and Orthopaedics, Ruijin Hospital, Shanghai Jiao Tong University School of Medicine, Shanghai, 200025, PR China; d State Key Laboratory of Ultrasound in Medicine and Engineering, Chongqing Medical University, Chongqing, 400016, PR China

**Keywords:** Functional tissue repair, Spatiotemporal modulation, Physical factors, Chemical factors, Responsive biomaterials

## Abstract

The biological characteristics and microenvironmental changes at each stage of tissue repair are intricately linked, maintaining a dynamic spatiotemporal equilibrium. Monotherapies often fail to effectively address complex microenvironmental disturbances, but combining multiple factors (e.g., physical and chemical) represents a promising approach to enhance tissue regeneration and repair. However, there is a lack of comprehensive reviews that explore the efficacy and mechanisms of combining physical and chemical factors to promote repair. A multidimensional understanding of the spatiotemporal specificity in tissue repair, coupled with the integration of spatiotemporal regulation principles into the design of smart-responsive biomaterials, is essential for regulating the local microenvironment and maintaining dynamic equilibrium. Based on this, this review first discussed the pathophysiological characteristics of spatiotemporal changes in tissue repair associated with several diseases. Second, the advantages and disadvantages of the current means of spatiotemporal modulation of physical and chemical factors commonly used for tissue repair were summarized. Finally, therapeutic strategies that utilize smart response materials to modulate these changes through physical and chemical factors are outlined. In addition, current challenges and future prospects for the use of smart-responsive biomaterials in spatiotemporal regulation of tissue repair were discussed.

## Highlights

• Emphasizing the importance of physical and chemical factors in spatiotemporal regulation of various diseases.
• Design and application of intelligent responsive biomaterials for spatiotemporal regulation in functional tissue repair.
• This review summarizes current strategies for regulating tissue repair through materials, providing multiple perspectives for future treatments of tissue repair.

## Graphical abstract

The role of smart responsive spatiotemporal-modulated materials mediated by physical and chemical factors in the spatiotemporal disorder phenomena of tissue repair in various disease types.

**Figure:**
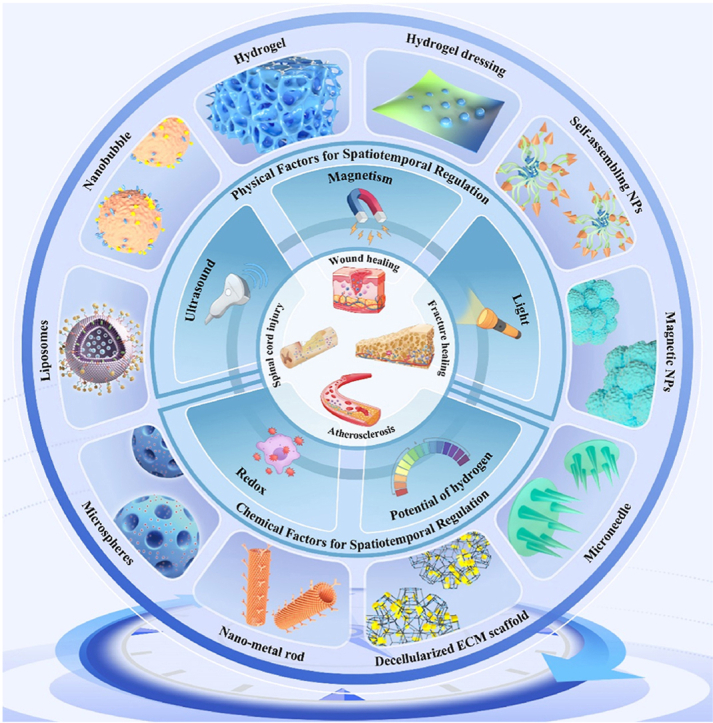

## 1 Introduction

Each stage of tissue repair exhibits distinct biological characteristics and microenvironmental changes, which create a phenomenon referred to as spatiotemporal specificity [1,2]. In a two-dimensional framework, temporal specificity corresponds to the horizontal axis, representing the distinct phases of tissue repair (time), which unfold through an intricate, interlocking process. In contrast, spatial specificity is depicted on the vertical axis, where the activity levels of cell-secreted factors and various microenvironmental components peak (space), significantly influencing the repair process and its outcomes [3]. Any factor-induced disruption in the spatiotemporal dynamics of tissue can affect the progression of tissue repair [4,5]. Generally, the tissue repair process is typically divided into three overlapping yet distinct phases: inflammation, proliferation, and remodeling [6]. The inflammatory phase forms the foundation of tissue repair. However, if the complex local pathological-immune response is not timely regulated, it may exacerbate tissue damage [7]. The proliferative phase is characterized by the rapid tissue formation, where oxygen supply [8], nutrient factors [9,10], and extracellular matrix reorganization [11] directly influence cellular proliferation and migration. During the remodeling phase, newly formed tissues mature and integrate into the host environment. However, cellular dysfunction and inadequate growth factors can impede effective tissue reconstruction [12]. Therefore, a comprehensive understanding of the spatiotemporal regulation in tissue repair progression will elucidate the underlying biological mechanisms and offer new insights for clinical treatment.

Biomaterials are biocompatible substances that can be implanted into the body for tissue repair therapies or diagnostic applications [[13], [14], [15]]. With advancements in biomedicine, a single biomaterial increasingly fails to meet the demands of tissue repair, leading to a burgeoning interest in developing biomaterials that respond to specific stimuli [16]. Responsive biomaterials are intelligent materials capable of releasing drugs, modulating the microenvironment, and providing support and protection at the injury site in response to specific stimuli [17]. Stimuli-responsive biomaterials can be categorized based on the type of stimulus into two main groups: physical and chemical stimulus-response. Physical stimuli include ultrasound, magnetic fields, and light. Ultrasound is characterized by precision and deep tissue penetration; magnetic fields enable facilitate targeted drug delivery; and light offers thermal effects and convenience [18,19]. Chemical stimuli include pH and redox conditions [[20], [21], [22], [23]]. Studies have demonstrated that anaerobic glycolysis in tumor cells produces large amounts of lactic acid, which acidifies the extracellular environment and enhances the response to pH-sensitive biomaterials [24].

With a deeper understanding of tissue repair, modulating the spatiotemporal dynamics of tissue repair has become an essential goal in designing smart responsive biomaterials. We have developed a bone spatiotemporal immunomodulatory hydrogel that can be remotely activated by ultrasound to release therapeutic agents at the peak of inflammation. This strategy modulates spatiotemporal disturbances and improves the local immune microenvironment, thereby promoting the repair of significant bone defects [4]. Chimeric Antigen Receptor (CAR) T-cell therapy may induce proinflammatory cytokine release supraphysiologic levels, leading to fever and multiorgan dysfunction [25]. Li et al. developed a temperature-sensitive hydrogel that intelligently detects and reduces suprathreshold levels of interleukin-6 (IL-6) during CAR T-cell treatment [26]. Therefore, harnessing the intelligent properties of responsive biomaterials to precisely regulate cell behavior and microenvironmental changes for tissue regeneration and repair is essential.

**Fig. 1:**
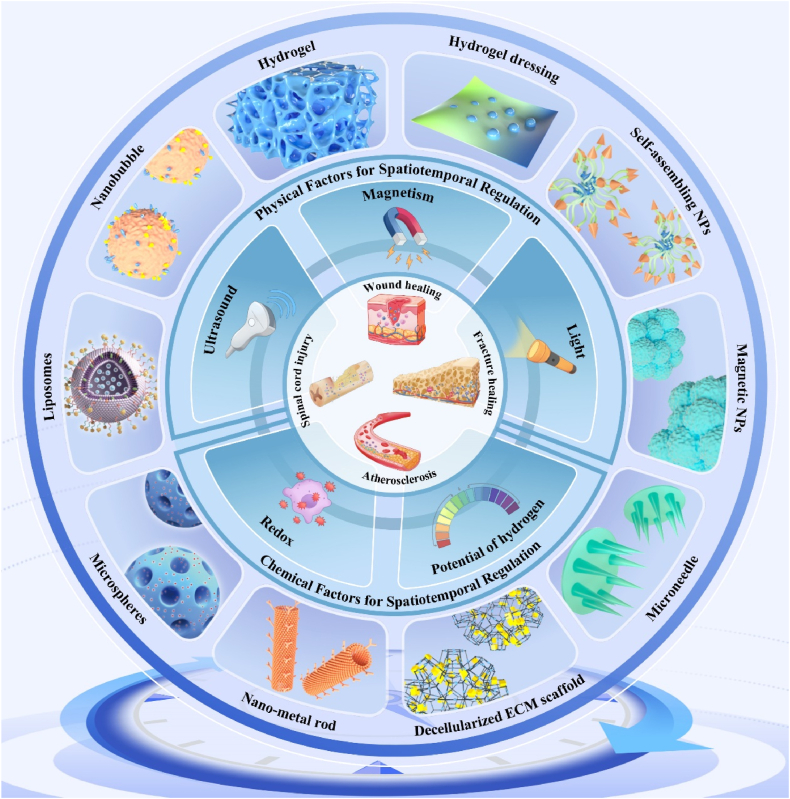
**Schematic illustration of smart responsive biomaterials for spatiotemporal modulation of functional tissue repair.** The mechanism diagram illustrates the role of multiple factors in the spatiotemporal disorder phenomenon of tissue repair in **v**arious types of diseases. According to the type of spatiotemporal regulation, it can be divided into intelligent responsive spatiotemporal regulation materials mediated by physical and chemical factors.

There have been numerous research studies and systematic reviews on smart responsive biomaterials for tissue repair [18,20,[27], [28], [29]]. This review serves as the first comprehensive and systematic compendium of research advances in spatiotemporal modulation through smart-responsive biomaterials. In this review, we explore the pathophysiological characteristics of spatiotemporal changes during functional tissue repair, followed by a discussion of smart-responsive biomaterials capable of modulating these dynamics. The article also briefly summarizes therapeutic strategies utilizing smart-responsive materials based on physical and chemical factors to modulate spatiotemporal changes in systemic tissue repair and discusses current challenges and prospects (Fig. 1). This knowledge may guide the development of advanced biomaterials capable of modulating spatiotemporal changes in tissue repair to address the therapeutic needs of various types of tissue repair.

## 2 Pathophysiological characteristics of spatial and temporal changes in functional tissue repair

As mentioned earlier, the spatiotemporal regulation of tissue repair relies on the coordination between unique biological features and microenvironmental changes within a specific spatiotemporal context. Tissue repair exists in a dynamic state of flux, with biological features and microenvironmental changes continuously interacting throughout the process. However, most research findings are limited to a particular stage of understanding. The tissue repair process can be broadly categorized into three phases: inflammatory, proliferative, and remodeling. It is characteristic of spatiotemporal regulation of tissue repair to regulate accordance with the distinct pathophysiological features of each phase [30,31]. Time and space are inseparable, and a multidimensional understanding of the spatial and temporal characteristics of tissue repair is crucial for effective repair. Next, we will describe the pathophysiological characteristics of spatiotemporal changes across various types of functional tissue repair.

### 2.1 Wound healing

Wound healing is a highly organized and dynamic process involving four phases: hemostasis, inflammation, proliferation and remodeling [32]. During this process, the requirements for wound healing change significantly over time, and the pathophysiological characteristics of each stage give rise to distinct therapeutic demands. Any deficiencies in addressing these needs may impede the body's ability to heal the wound effectively [33,34]. Adverse conditions such as hypoxia [35] and hyperglycemia [36] can disrupt this temporal transition, leading to the formation of chronic wounds. Additionally, various cellular and tissue dynamics during the proliferative and remodeling phases are critical for effective wound healing [6,37,38]. Therefore, it is essential to monitor the spatiotemporal kinetic changes of cells and their environment during tissue repair. On-demand spatiotemporal regulation of the tissue repair process is crucial for facilitating proper wound healing.

During the hemostatic phase, platelets aggregate at the injury site, triggering a cascade of coagulation reactions that culminate in thrombus formation [39]. Activated platelets release growth factors and cytokines, such as platelet-derived growth factor [40] and transforming growth factor-β [41], which recruit immune cell populations predominantly composed of neutrophils and macrophages, thereby initiating the inflammatory phase [42]. During the inflammatory phase (within 48 h of injury) neutrophils produce significant quantities of reactive oxygen species (ROS) and release neutrophil extracellular traps that engulf pathogens [33]. Macrophages secrete pro-inflammatory factors that promote skin barrier formation [43]. Within 2–3 days after injury, the number of macrophages peaks, ROS are progressively decline, and neutrophil extracellular traps disappear. Additionally, macrophages shift to an anti-inflammatory phenotype to promote tissue repair [44]. However, in the presence of adverse factors such as hypoxia and hyperglycemia, this spatiotemporal shift is disrupted, leading to the development of spatial disturbances such as elevated levels of ROS and acids, decreased cellular clearance, and aberrant pro-inflammatory macrophages, which exacerbate tissue damage and lead to chronic wound formation [45]. Studies have shown that dynamic changes in Notch signaling in wound macrophages are critical for wound healing. Early Notch expression in macrophages promotes their functionality during the inflammatory phase, whereas delayed regression of Notch signaling peaks (spatial disorganization) in late macrophages is an important contributor to increased wound inflammation and impaired healing [46]. Although an acidic pH is necessary for the early stages of acute healing, the optimal pH for the proliferation of keratinocytes and fibroblasts ranges from 7.2 to 8.3 [47]. The transition from an acidic to an alkaline microenvironment is one of the manifestations of spatiotemporal homeostatic properties of PH in the early and middle stages of wound healing. As inflammation and abnormalities are controlled, significant amounts of neovascularization, fibroblasts, and macrophages contribute to forming loose granulation tissue, signaling the transition of the repair process into the proliferative phase [48]. During the proliferative phase, epithelial cells fill the missing areas through epithelial-mesenchymal transition. Approximately seven days after injury, myofibroblasts facilitate wound contraction, initiating a carefully choreographed period of remodeling and repair that will last approximately one year [49]. During the remodeling phase, collagen and other proteins deposited in the wound undergo a continuous synthesis and degradation process to enhance the tissue's strength and integrity [50]. In wounds that heal smoothly, the ratio of type I protofibrillar collagen to type III collagen increases, shifting toward the composition found in normal skin, thereby achieving mechanical properties similar to those of healthy skin tissue [51].

**Fig. 2:**
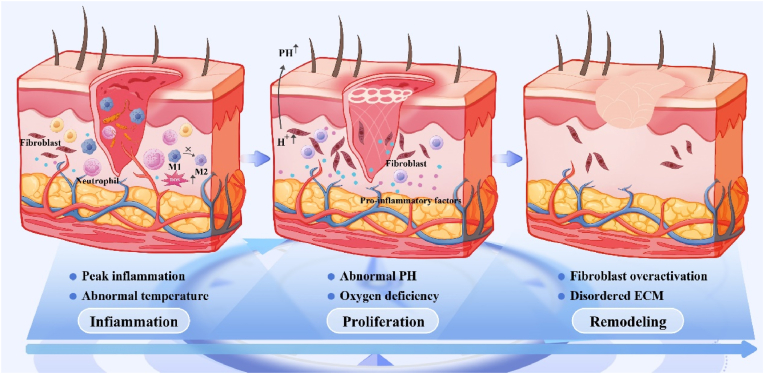
**Pathophysiological characteristics of spatial and temporal changes in wound healing.** During the inflammatory phase, persistent infections or inadequate blood supply cause wound pH to fluctuate into the alkaline range, which negatively impacts the healing process [47,231]; In late inflammatory phase, the disruption of the programmed transformation of M1 macrophages into the M2 exacerbates inflammatory infiltration [45]. Additionally, low skin temperature observed in infected wounds during the first two postoperative days is a critical factor impairing the transition to the repair phase [232]; During the repair phase, prolonged hypoxia inhibits essential processes (angiogenesis, re-epithelialization, and ECM synthesis), ultimately impairing wound healing [233]; During the remodeling, excessive activation of myofibroblasts, combined with impaired matrix remodeling, contributes to abnormal wound remodeling and promotes scar formation [234].

Therefore, we should focus on the spatiotemporal dynamics of multiple cells and tissues during tissue repair to ensure that the wound healing process proceeds appropriately. For example, as previously mentioned, during the late inflammatory phase (time), macrophages and neutrophils exhibit “brake abnormally” for various reasons (space). In the repair phase, slow angiogenesis and reduced stem cell repair function occur due to inadequate blood supply, oxygenation, and pH regulation. During the remodeling phase, abnormal fibroblast proliferation and disrupted stromal mechanics also play crucial roles. Thus, understanding these dynamics is essential for facilitating regular and orderly wound healing (Fig. 2).

### 2.2 Fracture healing

Fracture healing is a complex and highly coordinated process that involves temporal and spatial changes in various cellular and molecular signals [52,53]. The fracture healing process is typically classified into three primary phases: the inflammatory, bone repair, and stress remodeling [54]. Each stage is accompanied by a specific pathophysiologic process and has different treatment needs [55,56]. For example, immune responses during the inflammatory phase are critical for bone healing, especially pro- and anti-inflammatory transformation of macrophages [57]. Failure to effectively regulate these spatiotemporal factors is likely to result in delayed or non-healing fractures. Therefore, an in-depth investigation of the spatiotemporal dynamics of tissues and cells during fracture healing is essential for promoting routine fracture healing and repair.

Regardless of the fracture type, it is consistently accompanied by a robust inflammatory response [58]. Injury to soft tissue, cortical bone, cancellous bone, and bone marrow results in the rupture of bone microvessels and the formation of a hematoma [59]. Tumor necrosis factor α peaks one day after injury, promoting the recruitment of neutrophils and monocyte-macrophages, and returns to baseline within 72 h post-injury [60,61]. Abnormal spatial disturbances of tumor necrosis factor α after fracture (e.g., impaired expression on the day after injury and persistently high levels on the 3-day post-injury period) affect the entire process of fracture repair, which impairs the process of healing tissue repair and regeneration. Due to the interruption of blood supply, macrophages transition from aerobic respiration to glycolysis in response to local injury, activating pro-inflammatory macrophages (M1). Studies have demonstrated that inflammation mediated by M1-type macrophages peaks three days following bone injury, after which there is a transition to anti-inflammatory M2 macrophages [62]. The transformation of macrophages from a pro-inflammatory to an anti-inflammatory phenotype is crucial for facilitating the transition from the inflammatory phase to the repair phase of bone healing [63,64]. Our previous studies demonstrated that 24–48 h after bone injury, the immune system had a limited capacity for self-regulation, resulting in an inability to suppress inflammatory peaks timely (space). Additionally, modulating the excessive immune response during the inflammatory period restored immune microenvironmental homeostasis and promoted the repair of bone defects [4]. One day after osteotomy, a significant number of HIF1α-positive endothelial cells are expressed at both ends of the fracture in response to tissue hypoxia, while HIF1α signaling diminishes after three days [65]. Research demonstrated that during hypoxia, the overexpression of YAP1/TAZ was a significant factor contributing to the decreased coupling function between angiogenesis and osteogenesis (space) [66]. Metformin promotes H-vessel formation and accelerates the coupling of vascularization and bone repair by inhibiting YAP1/TAZ expression while upregulating HIF-1α expression in endothelial cells [67]. As the hematoma resolves, the body transitions into the bone repair phase. Undifferentiated mesenchymal stem cells (MSCs) in the central region of the fracture proliferate and differentiate into chondrocytes, forming a softer cartilage healing tissue [52]. Partial MSC differentiation of bone cortex into osteoblasts to form a ring of hard healing tissue [68]. Mature T and B lymphocytes accompany osteoblasts and osteoclasts, participating in the bone healing process through the RANKL-RANK-OPG system [69]. Subsequently, osteoclasts gradually resorb the cartilage matrix, while osteoblasts begin depositing calcium and forming primary trabeculae, marking the transition of fracture healing into the stress remodeling phase [52]. During remodeling, osteoclasts and osteoblasts collaboratively reconstruct the bone marrow cavity, repopulating hematopoietic tissue and restoring the original stress structure of the bone [70].

**Fig. 3:**
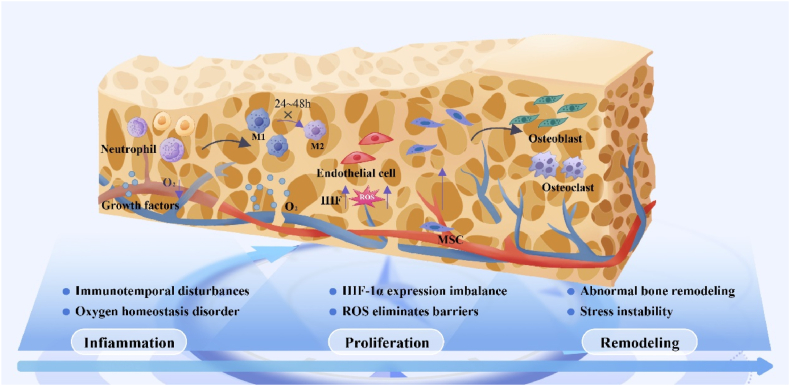
**Pathophysiological characteristics of spatial and temporal changes in facture healing.** Macrophage-mediated immune spatiotemporal disturbances occurring within 24–48 h after bone injury exacerbate the inflammatory infiltrate [2]; A prolonged hypoxic microenvironment significantly impairs vascularization at the site of bone defects, leading to failure of bone repair [[167], [168], [169]]; At the conclusion of the inflammatory phase, imbalanced expression of HIF-1α and impaired elimination of ROS hinder MSC recruitment and osteoblast activation [72,170,171]; During the repair phase, inadequate vascular establishment and biomechanical stress instability create a functional imbalance between the resorption of hard healing tissue by osteoclasts and lamellar bone deposition by osteoblasts, thus hindering effective skeletal remodeling [61].

In general, the phases of the fracture healing process occur sequentially, with immune cells and osteoblasts fulfilling their respective roles. However, the spatiotemporal regulation of fracture healing is influenced by various factors. Bacterial infections during the inflammatory phase (time) can induce a delayed peak of inflammation at the fracture site [71]. Controlling bacterial infection and modulating excessive inflammation levels (space) is critical for ensuring the initiation of tissue repair during fracture healing. In the typical fracture healing process, the upregulation of HIF-1α expression and reactive oxygen species (ROS) production during the early inflammatory phase serves as a crucial stress response that promotes the resorption of local necrotic tissues and hematomas. However, if the body fails to timely regulate HIF-1α expression and ROS production, it can reduce osteoclast activity and a diminished capacity for bone formation, ultimately resulting in delayed or non-healing fractures [72]. Therefore, we should investigate the spatiotemporal dynamics in tissues and cells during fracture healing to precisely regulate their disturbances and promote the normal tissue repair process (Fig. 3).

### 2.3 Atherosclerosis

Atherosclerosis is a chronic, progressive circulatory system disease that typically occurs in major blood vessels, such as the coronary arteries, significantly impairing blood supply to the heart [73]. The progression of the disease exhibits significant spatiotemporal specificity, characterized by both the advancement of disease stages and changes in plaque composition and local hemodynamics [74]. For instance, intima-vascular damage [75], the abnormal accumulation of low-density lipoprotein (LDL) [76], and changes in local pH [77] represent significant spatiotemporal factors in the progression of atherosclerosis. Thus, understanding spatiotemporal regulation in atherosclerosis can aid in optimizing therapeutic strategies and promoting tissue repair.

Normal arteries consist of a three-layered structure consisting of an outer membrane, a middle membrane, and an inner membrane (with blood flow) [78]. When blood vessels are chronically exposed to risk factors such as hyperglycemia [79] and hypertension [80], the endothelial barrier function of the arterial lining becomes disrupted, leading to the slow deposition of LDL in the inner membrane. As time progresses, rolling monocytes in the bloodstream migrate to dysfunctional endothelial cells, where they differentiate into macrophages, phagocytose lipids and progressively form an unstable core of atherogenic lipids [81]. It was found that macrophages were dramatically recruited to the site of injury and that smooth muscle cell proliferation accelerated as early LDL accumulation in the subendothelium reached a threshold; however, this recruitment and proliferation gradually declined at a later stage (after 10 weeks) [78,82]. Focusing on the spatial disorganization of aberrant macrophage recruitment is an important time for early spatiotemporal modulation of coronary atherosclerosis. Nitric oxide (NO) is a crucial signaling molecule essential for regulating cardiovascular diseases, and both elevated and diminished local concentrations can negatively impact the progression of these conditions [83,84]. However, in response to inflammatory stimuli, foam cells within plaques produce elevated levels of NO (>1 μM) through NO synthase, leading to oxidative, nitrative, and nitrosative stress [85]. In addition, atherosclerosis exhibits not only temporal and spatial characteristics during disease progression but also site-specific manifestations [74]. The hemodynamic characteristics of specific regions make the endothelium more vulnerable to mechanical stress injuries, thereby promoting plaque accumulation [86]. As the plaque progresses, localized alterations in blood flow exacerbate the damage and further accelerate the advancement of the lesion. When the plaque ruptures, the exposed lipid core interacts with clotting factors in the blood, triggering thrombosis and resulting in an acute coronary event [87].

**Fig. 4:**
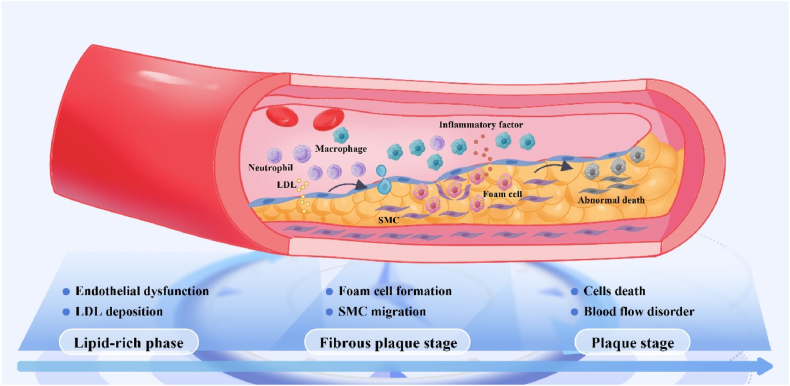
**Pathophysiological characteristics of spatial and temporal changes in atherosclerosis.** During the lipid-rich phase, the endothelial cell barrier function of the arterial endothelium becomes disrupted, leading to the slow deposition of LDL in the subendothelial space [79,80]; During the fibrous plaque stage, Foam cells subsequently secrete pro-inflammatory mediators that recruit immune cells (neutrophils, monocytes, and T cells) to the endothelium, thereby creating a positive feedback loop of inflammation and exacerbating atherosclerotic plaque formation [78]. Over time, the accumulation of lipids, abnormal smooth muscle cell death, and decreased pH and protein phosphorylation levels contribute to plaque instability [90,235]; During the plaque stage, the gradual expansion of atherosclerotic plaques disrupts blood laminar flow, triggering abnormal platelet activation and the initiation of the coagulation cascade, which promotes thrombosis and leads to progressive occlusion of the coronary vasculature [236].

The abnormal accumulation of LDL in the subendothelium may represent one of the earliest spatiotemporal disturbances in atherosclerosis, and this abnormality is considered a critical factor in initiating the pathophysiological progression of the disease. Acid-base homeostasis [88] and protein phosphorylation [89] are critical mechanisms in biological processes. During the progressive phase of atherosclerosis (time), multifactorial-induced decreases in pH and elevated protein phosphorylation levels hinder the protective functions of vascular endothelial cells (space) [90]. Therefore, comprehending the complex spatiotemporal properties during the progression of atherosclerosis is crucial for inhibiting excessive abnormalities at the injury site at specific time points, regulating spatiotemporal disorders, and ensuring orderly tissue repair in the body, ultimately aiding in the prevention and treatment of atherosclerosis (Fig. 4).

### 2.4 Nervous system

#### 2.4.1 Spinal cord injury (SCI)

SCI is a severe neurological disorder whose progression can be categorized into four distinct stages: acute, subacute, intermediate, and chronic phases [91,92]. The temporal and spatial characteristics of each phase play a crucial role in recovery from injury. For instance, during the acute phase, an adequately regulated neuroinflammatory response can facilitate repair, whereas an inappropriate inflammatory response may exacerbate spinal cord tissue damage [93]. In the subacute and intermediate phases, activated astrocytes contribute to recovery impairment by forming a neuroglial scar [94]. Therefore, an in-depth exploration of the dynamic changes in spatiotemporal regulation in SCI is crucial for optimizing treatment strategies and enhancing the effectiveness of repair.

The injury cascade initiated during the acute phase leads to ischemia, inflammatory cell infiltration, and cell death, with neuroinflammatory infiltration serving as a critical process that significantly impacts recovery from the injury [95]. While Appropriate inflammatory infiltration allows for rapid repair of damage, but neuroinflammatory disorders further destroy surviving spinal cord tissue and axonal demyelination [92]. Li et al. found temporal and spatial disturbances in neuronal focal death and apoptosis during early SCI. Neuronal apoptosis peaked one day after SCI, while focal death reached its peak three days later. Early and timely targeting of spatial disorganization of neuronal apoptosis is decisive for controlling focal necrosis resulting from subsequent neuronal injury. The knockdown of the microglial Hv1 proton channel inhibited neuronal apoptosis and the activation of NLRP3 inflammasomes, which promoted axonal regeneration and enhanced motor function recovery following SCI [96]. The translocator protein (TSPO) is associated with the activation of microglia and astrocytes and serves as a crucial biomarker for assessing neuroinflammatory activity [97]. Miu et al. longitudinally monitored the spatiotemporal changes of TSPO at the injury site in SCI rats using positron emission tomography. They found that neuroinflammation at the injury site peaked within two weeks and that its spatiotemporal kinetic alterations were closely correlated with the behavioral recovery of the SCI rats [98]. In the subacute phase, ischemia and inflammatory infiltration exacerbate the lysis of extracellular structures in the spinal cord, promoting the formation of cystic microcavities and the deposition of astrocytes [99]. Massively activated astrocytes, stimulated by neuroinflammation, act as negative regulators of recovery following SCI [100]. Ringless finger protein 1 (RING 1) is a protein of the RING family associated with a zinc-dependent DNA-binding structural domain [101]. Studies demonstrated that high expression of RING1 protein is an initiator of astrocyte proliferation and nerve fiber scar formation. During the acute injury period of SCI, astrocytes transcribed RING1 mRNA in large quantities and reached peak protein expression during the subacute period (day 3 post-injury) [102]. In the intermediate and chronic phases, as inflammation subsides, the body begins to regenerate myelin sheaths, reorganize blood vessels, and remodel neural circuits. Simultaneously, a substantial number of proliferating astrocytes differentiate and form a glial scar, which acts as a barrier to neural axon growth [103].

**Fig. 5:**
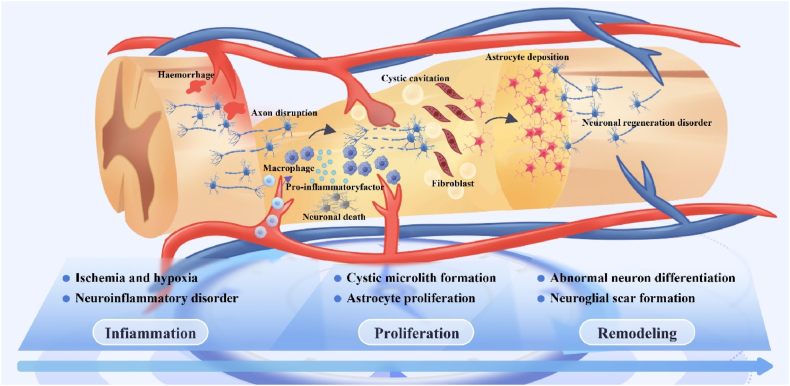
**Pathophysiological characteristics of spatial and temporal changes in SCI.** The injury cascade in the acute phase triggers excessive ischemia, inflammatory cell infiltration, cell death, and neuroinflammatory disturbances, which further exacerbate damage to surviving spinal cord tissues and lead to axonal demyelination [96]; During the subacute phase, spinal cord cells and extracellular structures are lysed, which leads to the formation of cystic microcavities and the deposition of astrocytes [99]; In the intermediate and chronic phases, as inflammation subsides, the organism initiates myelin regeneration, vascular reorganization, and remodeling of neural circuits. Concurrently, proliferating astrocytes differentiate to form a glial scar, which acts as a barrier to the growth of neural axons [103].

For SCI, the acute phase inflammatory cascade reaction process is closely linked to tissue repair, and the inability of the locally complex pathologic immune response (time) to adjust in time to suppress the acute immune peak (space) is a major cause of increased spinal cord tissue destruction and axonal demyelination. Additionally, the spatiotemporally disturbed pathomechanism underlying the extensive formation of neuroglial scarring during the intermediate and chronic phases is a major impediment to tissue repair. Therefore, it is essential to focus on exploring the temporal and spatial disturbance processes of pathophysiology during SCI and intervening in them to promote tissue repair (Fig. 5).

#### 2.4.2 Alzheimer's disease (AD)

AD is the most common neurodegenerative disease, and its pathogenesis is mainly due to the extracellular deposition of amyloid-β (Aβ) peptides and intracellular deposition of tau proteins in the cerebral cortex and hippocampus regions [104]. Research indicates that when the internal environment is unable to compensate for the excessive production of beta-site amyloid precursor protein cleaving enzyme-1 (BACE-1), it exacerbates the cleavage of amyloid precursor protein (APP) to produce Aβ peptides [105]. The high metabolic activity, high lipid content, and limited antioxidant defenses of neurons make them extremely susceptible to oxidative damage. Oxidative stress is an important cause of early progression in AD [106]. Copper, iron, and zinc are considered important trace metals that are essential for maintaining homeostasis in the internal environment, including the brain. Each gram of normal fresh brain tissue contains approximately 0.04 mg of iron [107]. However, excessive accumulation of trace metals can induce Aβ peptide aggregation and neuronal degeneration [108]. Therefore, it is crucial to focus on the treatment of spatiotemporal disorder phenomena in diseases with multiple systemic disorders. In addition, AD is similar to coronary atherosclerosis in that it is widely stimulated by pro-inflammatory signals and regulated by factors such as NO [109].

Recent studies have found that damage to the blood-brain barrier (BBB) is closely related to the onset of AD, and the destruction of the integrity of the BBB is considered an early biomarker of AD [110]. The BBB is a highly selective barrier composed of endothelial cells with tight junctions and a large number of transmembrane proteins (occludin, claudins, and junction adhesion molecule (JAM) proteins). Its cellular components are mainly endothelial cells, pericytes, basement membranes, and surrounding astrocytes [111]. By implementing the spatial partitioning effect, the BBB effectively isolates the intracranial microenvironment from the systemic circulation, maintaining the stability of the intracranial environment, protecting the brain from harmful substances and pathogens, and ensuring the normal functioning of brain functions [112]. Under pathological conditions, increased permeability of the BBB leads to reduced clearance of Aβ, accumulation of Aβ, and increased neurofibrillary tangles. More importantly, increased Aβ neurotoxicity further disrupts the structural basis, molecular regulation, and immune response of the BBB, forming a vicious cycle that exacerbates BBB damage [113].

Therefore, it is crucial to understand the spatiotemporal regulation of the progression of AD by clarifying the different spatiotemporal disorders, such as increased permeability of the BBB in the early stages of AD (space), accumulation of Aβ peptides and tau proteins in the brain (space), and oxidative damage to neurons and trace metal overload (space).

### 2.5 The importance of personalized spatiotemporal modulation

In summary, the importance of on-demand treatment at all stages of tissue repair is obvious. Blind, single anti-inflammatory or restorative treatments applied to tissues without proper evaluation often lead to detrimental and counterproductive effects. On-demand therapy is personalized and stage-specific, allowing the adjustment of therapeutic strategies to align with the patient's specific condition and pathological changes. This flexibility allows treatment regimens to be optimized for distinct pathological processes at various stages, thereby enhancing treatment outcomes. Thus, the identification of spatiotemporal disturbances in cellular and tissue dynamics specific to the wound healing phase is essential for the precise spatiotemporal regulation of functional tissue repair. Above, we have described some of the tissue repair spatiotemporal properties present in four diseases, aiming to emphasize the importance of focusing on spatiotemporal regulation in tissue repair progression. But What methods can be employed to target the modulation of the spatiotemporal properties of functional tissue repair? In the following section, we will describe the commonly used spatiotemporal modulation methods, including physical and chemical factors. Among them, physical factors represented by ultrasound, light, and magnetism have the advantages of instantaneous response, high penetration, and remote control. While chemical factors represented by pH and redox can adapt themselves to the microenvironment and promote cell activity and tissue repair.

## 3 Commonly used tools for spatiotemporal regulation

### 3.1 Physical factors

#### 3.1.1 Ultrasound (US)

US is a sound wave with a frequency higher than 20,000 Hz, with several physical properties, including good directionality, high frequency, short wavelength, and insignificant diffraction [114]. Depending on the frequency, direction of emission, and mode, various types of US can be generated, among which the most common are low-intensity pulsed ultrasound (LIPUS), Low-intensity focused ultrasound (LIFU), and high-intensity focused ultrasound (HIFU) [20,115]. The mechanisms of US primarily include mechanical, thermal, and cavitation effects. Mechanical effects can enhance tissue penetration and stimulate both the nervous system and cellular functions [116]; Thermic effect can cause a controlled rise in body temperature deep in the tissue [117]; Cavitation effect can produce a large number of small bubbles, triggering the vibration of the surrounding medium and produce high temperature and high pressure [12]. The mechanical effects of LIPUS and LIPU are known to promote cell regeneration, and open the BBB to enhance drug delivery [118,119]. Due to the absorption characteristics of biological tissues to ultrasound, so that part of the ultrasound energy into heat, causing the rise in tissue temperature, ultrasound is absorbed by the target tissue, the short-term increase in temperature can produce a strong thermal effect [120]. The immune system works at a higher temperature compared to normal body temperature [121]. Focused ultrasound-mediated temperature increase can improve the efficiency of the immune system and promote local tumor killing [122]. The primary factors affecting US transmission include the density of the medium, its viscosity, and the frequency of the ultrasound [123]. Due to the significant difference in acoustic impedance between air and liquids or solids, sound waves encounter difficulty in transitioning from air to liquids or solids, as well as from liquids or solids to air [124]. Therefore, the frequency and intensity of US need to be adjusted accordingly for different biological tissues with different US uptake.

The advantages of US in spatiotemporal modulation are primarily reflected in its tunable characteristics and precise mechanism of action. By adjusting the frequency, power, and irradiation time, US significantly influence the tissue repair process at specific time points. Additionally, the mechanical, thermal, and cavitation effects of US allow for targeted interventions tailored to different cellular and microenvironmental conditions (space). For instance, during the inflammatory phase, US enhances cellular permeability, improving drug delivery efficiency [125,126]; During the repair phase, local temperature elevation promotes blood circulation [127] and enhances cellular function [128]. Such spatiotemporal modulation capabilities make US an ideal treatment for optimizing the tissue repair process and enhancing efficacy.

#### 3.1.2 Light

Light is non-invasive and spatiotemporally specific, garnering widespread attention for its ability to precisely regulate tissue repair by adjusting parameters such as wavelength, power, and irradiation duration [129]. According to the triggering wavelength, light can be categorized into ultraviolet light (∼380 nm), visible light (380–780 nm) and infrared light (780 nm∼). The depth of light propagation depends on the output power, wavelength and the water content in a particular area. The longer the wavelength, the deeper the depth of penetration; the higher the power, the deeper the depth of penetration, and the more heat is produced [130]. However, due to the transmission limitations of light, it is primarily used for disease modulation at more superficial sites [131]. High-energy lasers (980 nm) represent a more advanced technology compared to other light therapies and can penetrate deeply into tissues, such as bone surfaces, to promote deep tissue repair [132]. Furthermore, doping photosensitive functional groups to enhance light energy absorption at deeper sites represents a novel approach for achieving deep-level modulation of light, including the formation or degradation of materials, contraction or expansion of networks, and controlled drug release [133]. Depending on the properties of the biomaterial, light-based stimulation can be finely tuned to elicit desired responses and regulate cell–material interactions [134]. Photothermal therapy (PTT) and photodynamic therapy (PDT) are emerging applications of photodynamics [135]. When stimulated by light at specific wavelengths, certain materials can convert light energy into heat energy, enabling locally controllable photothermal conversion, which can be applied to temperature-responsive drug delivery [136]. When different wavelengths of light act on tissues, light energy will be absorbed to different degrees and converted into heat energy [136]. Low power light irradiation can produce a slight thermal effect, stimulate tissue metabolism, improve blood circulation, promote wound healing or inflammation dissipation [137]. High power light irradiation will instantly cause tissue coagulation, carbonization to achieve minimally invasive surgery to stop bleeding or lesion removal [138]. Another light-responsive approach derived from this involves photosensitizing compounds. These photosensitizing components are activated by specific light excitation and interactions with oxygen, generating singlet oxygen and high concentrations of ROS, which exert a cytotoxic effect [139].

Light offers the distinct advantage of spatial and temporal regulation. By adjusting parameters such as wavelength, power, and irradiation time, specific biological responses can be activated at various stages of tissue repair (time), ensuring precise control over target tissues or cells (space). The high spatial resolution of light allows for selective modulation at different tissue levels, which, combined with precise temporal regulation, facilitates intervention in key cellular activities and microenvironmental changes throughout the disease process. Thus, the non-contact and transient response properties of light establish it as a crucial tool for optimizing therapy across different time and spatial.

#### 3.1.3 Magnetism

Magnetism combines the advantages of US and light, with instantaneous response, high penetration, remote control and high controllability, it is one of the most promising physical means [140]. Magnetically responsive biomaterials represent a class of materials that exhibit a high responsiveness to magnetic fields. When subjected to a specific external magnetic field, these materials can rapidly and precisely alter their physical or chemical properties, including magnetically induced orientation, mechanical stimulation, and magneto-thermal effects [141]. Magneto-responsive biomaterials usually consist of biocompatible materials and magnetic components, where the magnetic responsiveness is mainly derived from the doping with magnetic nanoparticles (MNPs) [142]. MNPs are nanomaterials with superparamagnetic properties and particle sizes of about 100 nm, nanoparticles that can be manipulated by external magnetic fields [143]. The advantage of MNPs is that their position can be controlled by applying an alternating magnetic field that does not adversely affect the body [144]. The application of a localized magnetic field allows the MNPs to reach the target site at precisely [145]. Immune site modification of MNPs improves target enrichment and facilitates disease diagnosis and treatment [146]. In addition, ferromagnetic nanoparticles possess peroxidase-like activity, enabling catalytic effects similar to those of horseradish peroxidase, which may further aid in therapeutic functions [147].

Like light and ultrasound, Magnetism, offers significant advantages for spatiotemporal regulation. By applying an alternating magnetic field (kHz-MHz), it can coordinate both temporal and spatial dimensions to precisely control interventions in specific pathological environments at various stages of disease progression (time) while also regulating peak moments of cellular activity by targeting specific spatial regions (space). This approach provides more accurate interventions for tissue repair. Consequently, by precisely regulating the spatial dimension of peak cellular activities at specific times and across different cell types, MNPs avoid temporal and spatial disturbances, effectively supporting the orderly repair of tissues. In addition, MNPs-mediated magnetic hyperthermia therapy (MHT) has recently emerged as a new strategy for thermotherapy, and MTH can be used as an adjuvant therapy to radiotherapy in the treatment of malignant tumors, as it induces cancer cell death at lower temperatures (41–46 °C) without damaging normal cells.

#### 3.1.4 Limitations of physical factors

While physical factors offer significant advantages in tissue repair and regeneration, they also have certain drawbacks. For instance, US is limited by acoustic impedance, light is restricted by penetration depth, and the effectiveness of magnetic fields is influenced by the specific conditions under which they are applied. In contrast, chemical factors possess site-specific characteristics, such as pH and redox differences, in abnormal biological environments. These characteristics can be leveraged to modulate the local microenvironment and enhance cellular activity, thereby more precisely influencing biological responses and tissue repair processes. Among the various chemical factors, pH and redox state are the most prevalent and critical influences. Therefore, we will next focus on the importance of these two chemical factors (biological) in tissue repair and their potential applications.

### 3.2 Chemical factors

#### 3.2.1 PH

Changes in pH are commonly used in the organism to trigger cellular and biological reactions [148]. For example, a decrease in pH from alkaline to acidic is critical for successful healing and also influences infection control, antimicrobial activity, oxygen release, angiogenesis, protease activity, and bacterial virulence [149,150]. Given the widespread pH fluctuations within tissues—especially under pathological conditions—the development of pH-responsive biomaterials has become a prominent area of research. PH-responsive biomaterial can respond to changes in the pH of a solution and thus change its structure and properties, and its responsiveness is mainly derived from polymers with ionizable acidic or basic groups [151]. Cationic and anionic polymers are two commonly used polymers [152]. Cationic polymers transition from hydrophobic to hydrophilic as the pH decreases, while anionic polymers shift from hydrophilic to hydrophobic under the same conditions [153]. Therefore, the properties of pH-responsive materials change with variations in ambient pH, including acid-induced swelling, alterations in solubility, modifications in charge, and cleavage of covalent bonds [154]. Based on these altered properties, pH-responsive biomaterials are utilized in drug delivery, protein adsorption, antimicrobial activity, and pH regulation.

Therefore, pH changes in the microenvironment are not only central to the spatiotemporal disruptions of tissue repair but also serve as a crucial mechanism for guiding the healing process to proceed in an orderly manner within a defined spatiotemporal framework. On the one hand, abnormal pH changes can be precisely harnessed to regulate specific pathological conditions (space) at various stages (time), ensuring that cellular and microenvironmental activities peak appropriately to restore the spatiotemporal balance of tissue repair. On the other hand, monitoring local pH provides insight into the spatial and temporal progression of tissue repair, guiding the development of novel therapeutic strategies.

#### 3.2.2 Redox

Redox reactions are prevalent in biological systems, and disruptions in redox balance are closely linked to the progression of numerous diseases. ROS and glutathione (GSH) play essential roles in modulating cellular function and disease pathogenesis [155,156]. Changes in the redox state of the cellular microenvironment induce the production of redox molecules, including free radicals. These molecules, owing to their unique biological properties, can readily cross the cell membrane, directly influencing the intracellular redox balance and thereby impacting cellular metabolic processes [157]. In pathological conditions, disturbances in the tissue redox system can result in inflammation, cellular damage, and disruptions to homeostasis [158,159]. For example, tumor tissues exhibit elevated levels of ROS and GSH compared to healthy tissues. Specifically, GSH levels in tumor cells are more than four times higher than those in healthy cells, indicating that tumor tissues are suitable targets for therapeutic interventions [160]. The redox responsiveness of biomaterials primarily depends on alterations in chemical bonding. Upon exposure to the specific redox environment, these chemical bonds are broken, resulting in either the degradation of the biomaterial or the release of the drug, thereby facilitating therapeutic functions [161].

Redox imbalance, an abnormal pathophysiological characteristic within the body's internal environment, manifests as a phenomenon of spatiotemporal disorder. Therefore, the elimination of abnormal redox states and the targeting of pathological sites through redox-based interventions show promise for the spatiotemporal regulation of tissue repair.

### 3.3 Expansion of common tools for spatiotemporal regulation

Both physical and chemical factors are indispensable tools for achieving spatiotemporal regulation. However, as previously mentioned, each modality presents inherent limitations. For instance, US is limited by acoustic impedance; the depth of light penetration is constrained; the effectiveness of magnetic fields depends on the specific conditions of application. Their limitations often lie in the universality of their effects, their ability to penetrate biological tissues, or their insufficient precision in directly regulating specific molecular events. And the modulation of microenvironmental pH and redox states faces challenges related to low precision, difficulties in evaluating effects, and unpredictability regarding toxicity and adverse effects. The initiation of chemical regulation often depends on intrinsic biochemical environmental changes, which may result in slower response times, making it difficult to precisely control temporal and spatial accuracy. Additionally, off-target effects and unpredictable toxicity in complex biological environments pose inherent challenges. It is precisely these challenges faced by physical and chemical factors that highlight the profound complementarity between them. Therefore, only by seamlessly integrating the two and promoting the synergistic advantages and mutual compensation of their disadvantages can efficient, precise, and safe spatiotemporal regulation be achieved. Biomaterials offer a robust platform to address the inefficiencies and lack of precision in single physical or chemical modalities. They amplify the advantages of these factors, optimize therapeutic effects, and enhance their comprehensive role in tissue repair. Consequently, employing spatiotemporal modulation in conjunction with the properties of biomaterials to regulate the local microenvironment and direct stem cell differentiation represents a significant advancement in the field of tissue repair and regeneration. In the following section, we will discuss the application of spatiotemporally regulated smart-responsive biomaterials in tissue repair, focusing on both physical and chemical factors.

## 4 Smart responsive biomaterials for spatiotemporal modulation

As previously noted, physical factors such as US, light, and magnetism, as well as chemical factors like pH and redox, offer substantial advantages in regulating tissue repair. However, significant limitations remain when these factors are used individually. For example, US, light, and magnetic modulation often exert effects over relatively broad areas, making it challenging to enhance their effects on specific tissue or cell types; At the same time, regulating pH or redox homeostasis at abnormal sites may provoke localized overreactions, potentially worsening tissue damage and hindering effective wound repair. Currently, a large number of responsive biomaterials are being used in clinical research experiments, such as pH-responsive cyclodextrin nanocarriers (CRLX101), tumor-targeted silica nanoparticles, glucose-sensitive insulin (MK-2640), SEL-212 [17]. Therefore, it is crucial to focus on developing biomaterials that can respond to physical and chemical factors that control the timing of tissue repair. In the following sections, we will explore specific applications of smart responsive biomaterials in tissue repair, primarily based on physical and chemical factors.

### 4.1 Physical factors responsive materials for spatiotemporal modulation

Materials responsive to physical factors offer the advantage of on/off control and the ability to precisely regulate the timing of material activation. However, the variability in the degree of spatiotemporal disorganization among individuals often limits the efficiency of materials. In the following sections, we describe the spatiotemporal modulation of tissue repair by materials responsive to physical factors, focusing on US, light, and magnetism.

#### 4.1.1 US-responsive materials

**Fig. 6:**
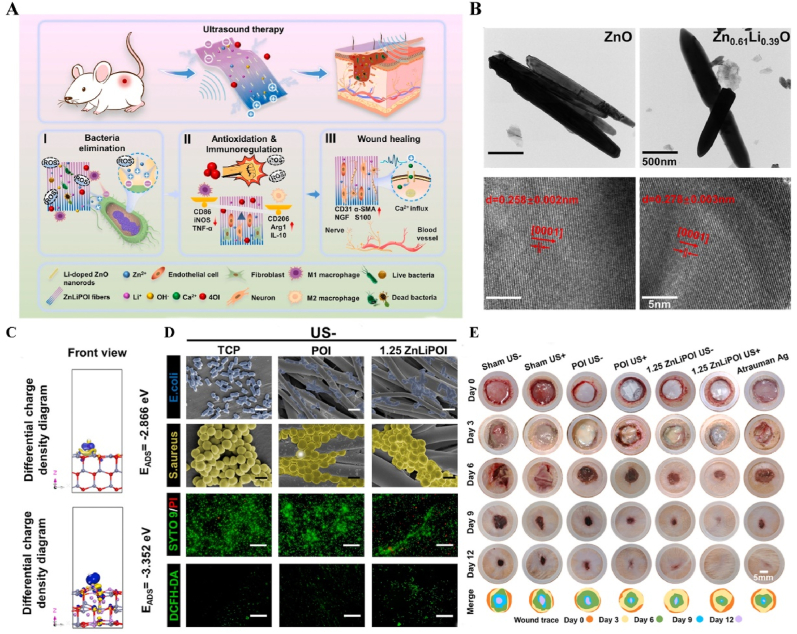
**US-responsive biomaterials for spatiotemporal modulation in infected wound healing.** A) Schematic diagram of US-responsive piezoelectric microfibers promoting wound healing. B) TEM images of ZnO and Zn_0.61_Li_0.39_O. C) Differential charge density diagram and adsorption energy to OH. D) The morphology of each group of colonies. E) Wound healing progress in different groups after surgery [165]. Reproduced with permission. Copyright 2025, Elsevier.

**Fig. 7:**
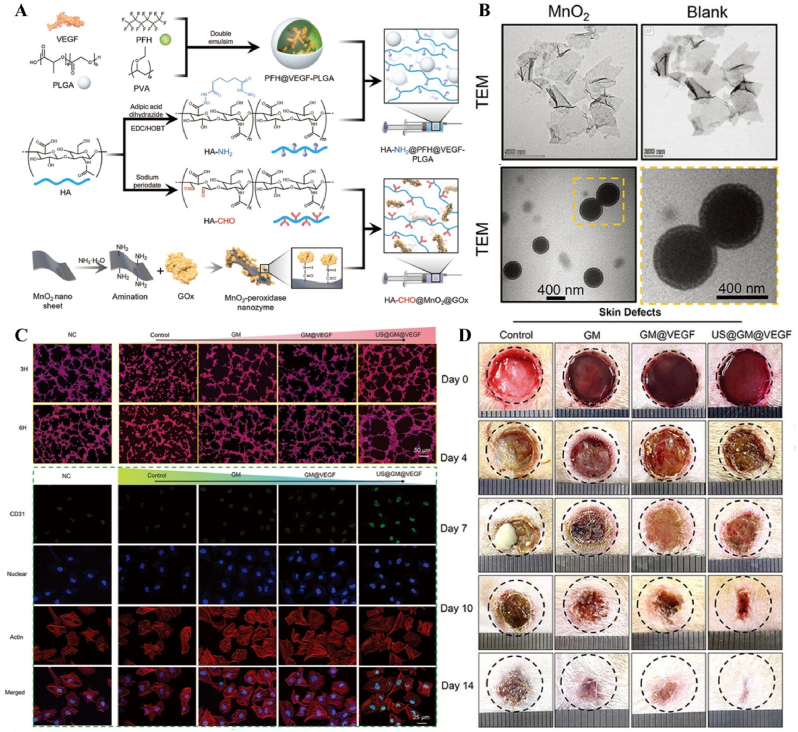
**US-responsive biomaterials for spatiotemporal modulation in diabetic wound healing.** A) Schematic diagram of the preparation of VEGF@PFH-PLGA NBs and MnO2@GOx. B) TEM images of MnO_2_ nanosheet and VEGF-PLGA NBs. C) Confocal images of vascular tube-formation and immunofluorescence images of CD31 with different groups. D) Wound healing progress in different groups after surgery [166]. Reproduced with permission. Copyright 2024, Wiley.

US-responsive materials show unique potential in the spatiotemporal modulation of disease progression [162]. Early infection is a major factor contributing to delayed wound healing and impaired vascular development [163]. Although an increase in ROS can be detrimental to bacteria, excess ROS during the inflammatory phase can impair normal mitochondrial function in cells, resulting in local spatiotemporal disturbances [164]. Maintaining a spatiotemporal balance between the beneficial and detrimental effects of ROS is critical for effective disease management. In one study, researchers developed a novel electroactive composite device (ZnLiPOI), which consists of lithium-doped ZnO/PLLA microfibers coated with the antioxidant 4 octyl itaconate (4OI), for wound healing. Early in the disease (time), US promoted the release of ROS from ZnO to kill bacteria (space); in the middle stage, antioxidant release promoted macrophage reprogramming and improved the immune microenvironment; in the late stage, Zn^2+^ and lithium ions were slowly released and cells were recruited through piezoelectric effect to promote wound recovery [165] (Fig. 6). Targeting spatiotemporal disorders in disease is crucial for effective regulation, and sensing the abnormal microenvironment serves as a key indicator of the spatiotemporal regulation of disease. We designed an injectable hyaluronic acid (HA) hydrogel encapsulating visualized nano-enzymes and therapeutic nanomicrobubbles (US@GOx@VEGF hydrogel). This design rapidly consumed and visualized glucose levels in the microenvironment *via* glucose oxidase@manganese dioxide (Gox@MnO_2_). Upon detecting a decrease in local glucose levels, the precise release of vascular endothelial growth factor (VEGF) was activated by US, promoting rapid blood vessel formation and accelerating repair [166] (Fig. 7).

**Fig. 8:**
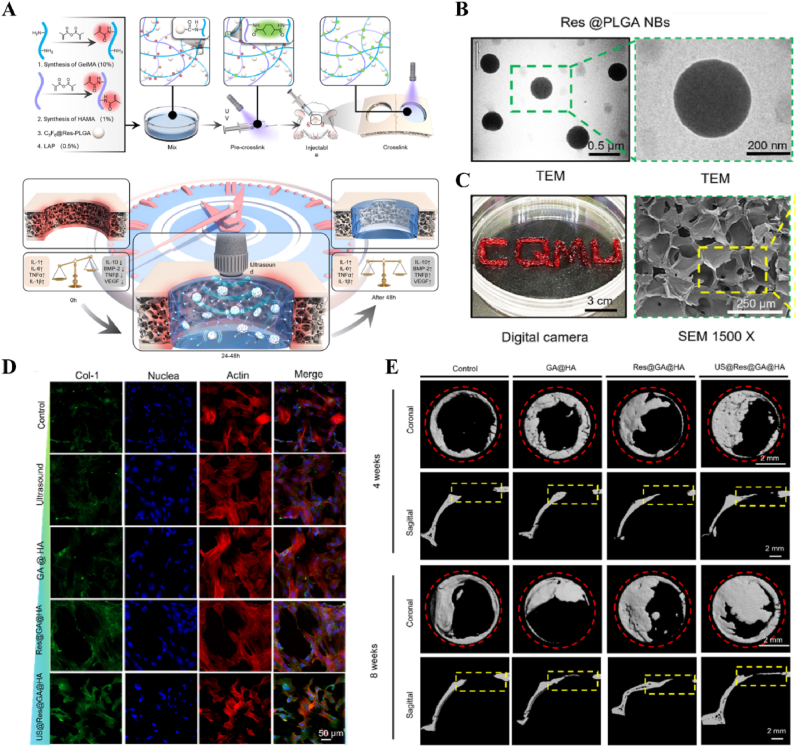
**US-responsive biomaterials for spatiotemporal modulation in bone defects.** A) Schematic diagram of the spatiotemporal interference process of UCE hydrogel regulating bone immunity. B) TEM images of Res@PLGA NPs. C) SEM images of UCE hydrogel. D) Image of BMSCs after immunofluorescence staining of Col-1. E) Micro-CT reconstruction of the calvarial defects [4]. Reproduced with permission. Copyright 2023, Elsevier.

**Fig. 9:**
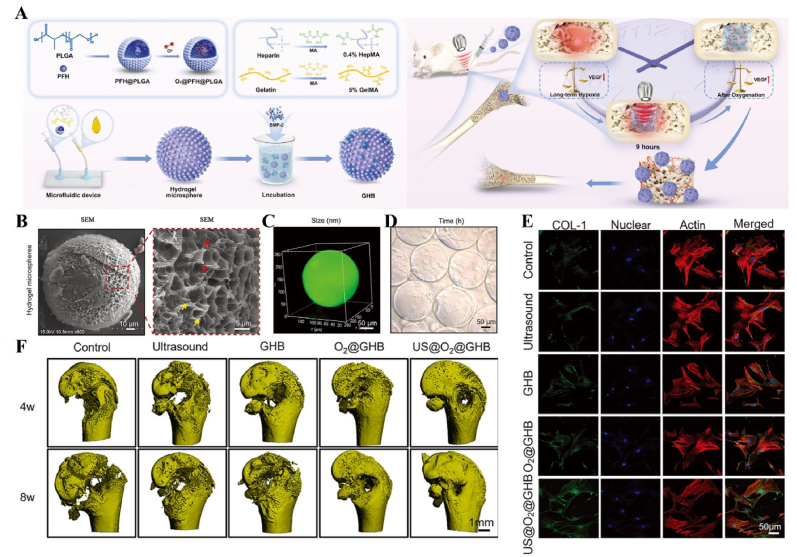
**US-responsive biomaterials for spatiotemporal modulation in large bone defects.** A) Schematic diagram of application of spatiotemporal hydrogel microspheres US@O2@GHB. B) SEM images of hydrogel microspheres. C) Images of hydrogel microspheres under confocal microscope. D) Images of hydrogel microspheres under light microscope. E) Image of BMSCs after immunofluorescence staining of Col-1. F) Micro-CT reconstruction of the femur [169]. Reproduced with permission. Copyright 2024, Wiley.

**Fig. 10:**
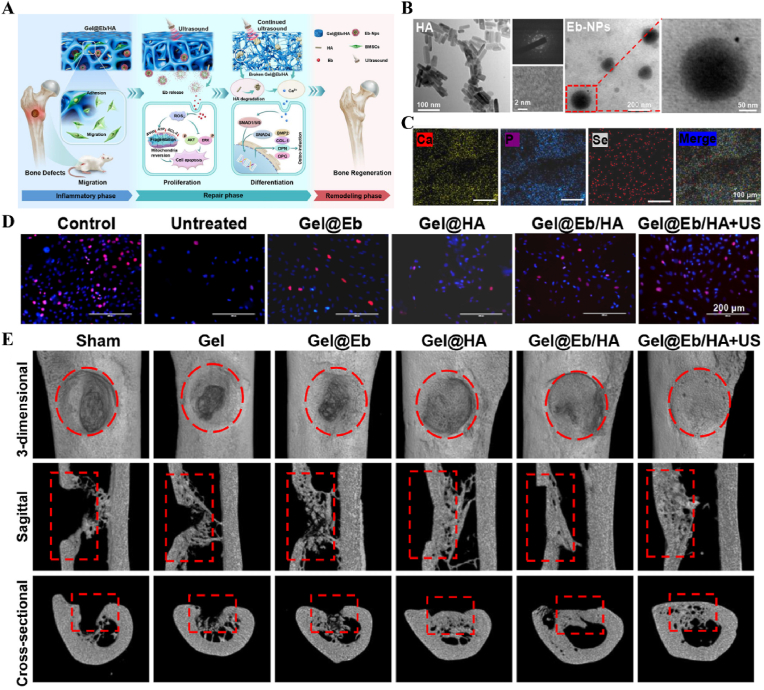
**US-responsive biomaterials for spatiotemporal modulation in bone defects.** A) Schematic diagram of US responsive hydrogel promoting multi-stage bone regeneration. B) TEM images of HA and Eb-NPs. C) EDS mapping of element distribution. D) EdU staining assay images of BMSCs. E) Micro-CT reconstruction of the femur [172]. Reproduced with permission. Copyright 2024, Elsevier.

Immunotemporal disturbances, especially within the first 24–48 h post-injury, are significant contributors to the failure of bone repair [2]. In response to this spatiotemporal alteration, we developed an US-triggered jet hydrogel containing RES-loaded PLGA nanobubbles (US@RES@GAHA). The release of RES was initiated by US on day 2 post-bone injury to inhibit the peak of the M1-mediated immune response, restore spatiotemporal homeostasis at the site of the injury, and promote bone repair [4] (Fig. 8). In addition to immune disorders, oxygen deficiency [[167], [168], [169]], low secretion of osteogenesis-related factors [170] and impaired bone marrow-derived stem cells (BMSCs) differentiation [171] are significant barriers to bone healing following an inflammatory peak. Accurate supplementation of oxygen to regulate the disruption of the spatiotemporal oxygen balance is essential for bone tissue regeneration. We successfully embedded oxygen-carrying nanobubbles, prepared through double emulsification, into the macromolecular network of GelMA/HepMA microspheres using microfluidics and constructed spatiotemporally controlled hydrogel microspheres by non-covalently binding bone morphogenetic protein 2 (BMP2). US-triggered cavitation hydrogel microspheres delivering oxygen at 9 h modulated local oxygen balance to promote vascularized osteogenesis and exhibit good angiogenic and osteogenic capacity, providing a new strategy for the clinical treatment of large bone defects [169] (Fig. 9). Another study synthesized a sodium alginate hydrogel (Gel@Eb/HA) composed of ebselenoline (Eb) and hydroxyapatite as the primary components. In the early stage of repair, Eb was rapidly released by US to alleviate oxidative stress. In the late stage of repair, Gel@Eb/HA underwent ultrasonic degradation to release a substantial amount of Ca^2+^, promoting bone formation [172] (Fig. 10).

**Fig. 11:**
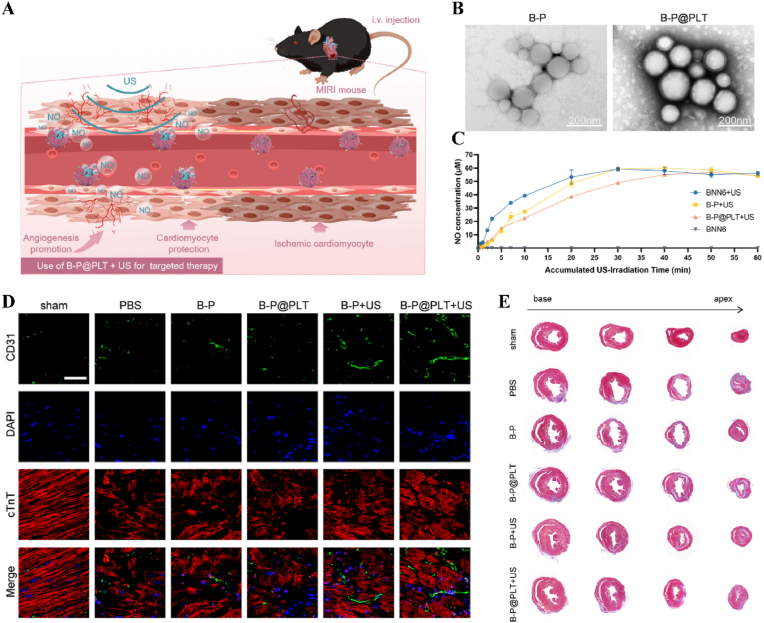
**US-responsive biomaterials for spatiotemporal modulation in myocardial ischemia reperfusion injury.** A) Schematic diagram of US triggering B-P@PLT promoting angiogenesis and protecting cardiomyocytes. B) TEM images of B-P and B-P@PLT. C) The NO release from each group under US. D) Image of endothelial cells after immunofluorescence staining with CD31. E) Masson trichrome staining of cardiac [173]. Reproduced with permission. Copyright 2023, Wiley.

**Fig. 12:**
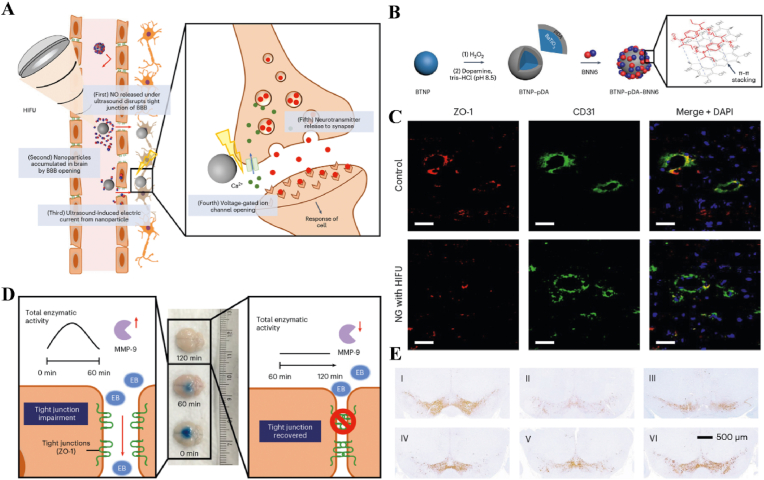
**US-responsive biomaterials for spatiotemporal modulation in AD.** A) Schematic diagram of US-responsive piezoelectric nanoparticle for neural stimulation. B) Scheme of preparation of the nanoparticle. C) Image of midbrain sections after immunofluorescence staining with ZO-1, CD31 and nuclei. D) Accumulation of EB over time in brain tissue. E) Images of TH in SNc 5 days after perfusion [174]. Reproduced with permission. Copyright 2020, Springer Nature.

In addition, US has a long-range penetration effect that promotes the localized release of payloads, which can be applied to the spatiotemporal modulation of circulatory-related diseases. Impaired NO production exacerbates myocardial ischemia-reperfusion injury (MIRI). N, N′-di-sec-butyl-N, N′-dinitroso-1,4-phenylenediamine (BNN6) serves as an ultrasound-sensitive NO donor, capable of the artificial and controlled release of NO upon ultrasound stimulation. BNN6 nanoparticles constructed on the basis of platelet membrane encapsulation targeted the myocardial region and released NO in response to US, thereby restoring endothelial cell function [173] (Fig. 11, Table 1). Due to the large bone structures surrounding the cranium and spinal cord, US signals are difficult to transmit. Therefore, US-responsive biomaterials are currently mainly used to open the BBB to promote drug accumulation in the nervous system. A nanoparticle composed of BNN6 and piezoelectric barium titanate (BTNP) coated with polydopamine (pDA) (BTNP–pDA–BNN6) can effectively open the BBB and alleviate PD symptoms. Under US stimulation, this system releases NO, temporarily disrupting the tight junctions of the BBB, delivering BTNP–pDA nanoparticles, and stimulating nerves through voltage-gated ion channels to improve PD symptoms [174] (Fig. 12).

**Table 1:** US-responsive biomaterials for spatiotemporal modulation in tissue repair.

Biomaterial design	US parameters	Application	Effects	Outcomes	Ref
Li-doped ZnO/PLLA microfibers with antioxidant 4OI coatings	early 1 MHz	Infected wound healing	The increase in ROS released by ZnO and Li	The inhibition of bacterial growth, improvement of the immune microenvironment and increase in cell migration, epidermal growth and angiogenesis treated with ZnLiPOI.	[165]
	1.5W cm^2^ 15min				
	late 1 MHz		Increased release of 4OI antioxidants, Zn^2+^ and Li^+^		
	0.3W cm^2^ 1min				
HA hydrogels encapsulating visualized GOx@MnO2 and VEGF-PFH-PLGA	650 kHz 1W cm^2^ 1min	diabetic wound healing	Precise release of VEGF	Visualized glucose depletion and precisely released VEGF to promote wound angiogenesis and accelerate repair treated with US@GOx@VEGF hydrogel	[166]
GelMA/HAMA hydrogels loaded with RES@PLGA nanoparticles	650 kHz 1.5W cm^2^ 1min	Bone defects	The released of RES caused by US stimulated to inhibit peak inflammation	Improvement of immune spatio-temporal disorders, maintenance of immune microenvironmental homeostasis and promotion of bone repair by UCE hydrogel	[4]
GelMA/HepMA microspheres containing oxygen-carrying nanobubbles and BMP2.	650 kHz 1.5W cm^2^ cm^2^ 1min	Bone defects	The rapid-released of oxygen caused by US stimulated and slow-released BMP2	Microsphere system improved the disturbance of oxygen balance in space and time, promoted vascularization and osteogenesis	[169]
Sodium alginate hydrogel with Eb and hydroxyapatite	1.5 MHz 0.4 Mpa 10 min	Bone defects	Rapid-released of Eb for ROS scavenging and slow-released Ca^2+^ for osteogenic differentiation of BMSCs	Ultrasonic stimulation of Gel@Eb/HA hydrogel alleviated oxidative stress and promotes osteogenic differentiation	[172]
Platelet membrane-encapsulated PLGA nanoparticles containing BNN6	1 MHz 1W cm^2^ 5 min	Myocardial ischemia/reperfusion injury	The released of NO caused by US stimulated to rejuvenation of endothelial cells	Tail vein injection of B-P@PLT targeted myocardial regions to reduce apoptosis, promote angiogenesis and improve cardiac remodeling	[173]

#### 4.1.2 Light-responsive materials

**Fig. 13:**
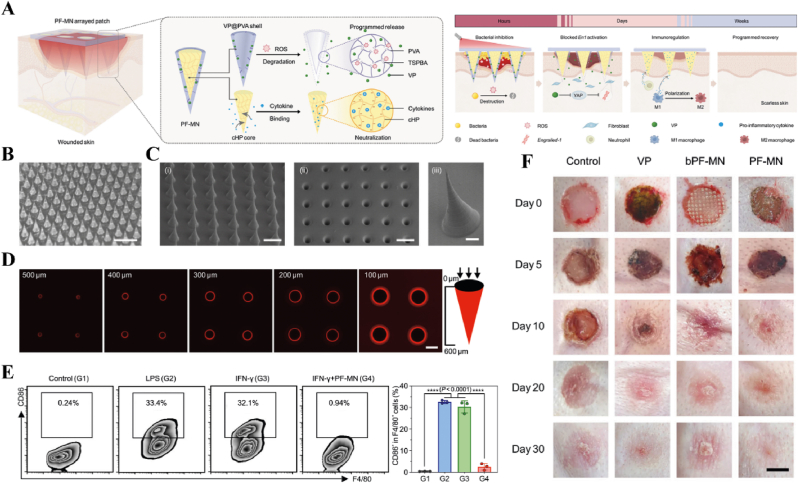
**Light-responsive biomaterials for spatiotemporal modulation in wound healing.** A) Schematic diagram of scarless wound healing process programmed by PF-MNs. B) Photograph of the PF-MNs. C) SEM images of PF-MNs. D) Confocal microscope images of PF-MN rhodamine staining. E) Flow cytometry of M1 macrophages. F) PF-MN-mediated wound healing on rabbit ears [178]. Copyright 2023, Springer Nature.

**Fig. 14:**
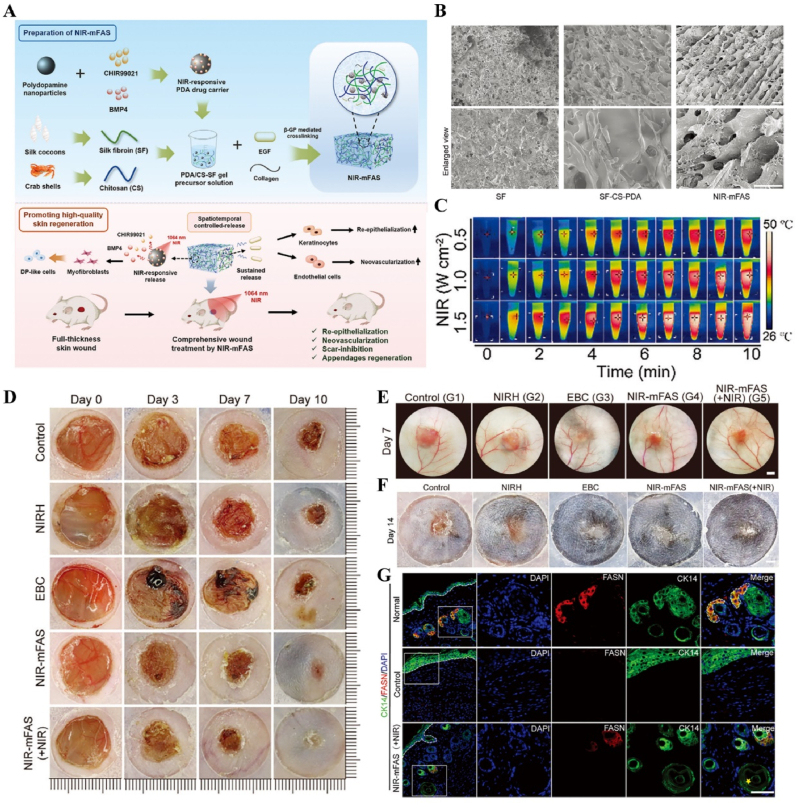
**Light-responsive biomaterials for spatiotemporal modulation in wound healing.** A) Schematic diagram of the preparation process of NIR-mFAS and its mechanism of promoting wound healing. B) SEM images of SF, SF-CS-PDA and NIR-mFAS. C) Real-time thermal images of NIR-mFAS. D) Wound healing progress in different groups after surgery. E) Photographs of the vascular network in different groups on day 7. F) Photographs of neogenic hair. G) Immunofluorescence staining of hair follicles and sebaceous glands [179]. Reproduced with permission. Copyright 2024, Wiley.

Light, as a flexible and non-invasive tool, can be adapted to the biological demands of different disease stages by varying its wavelength and intensity [175,176]. This fine-tuning allows photoresponsive materials to optimize the repair process by flexibly stimulating or inhibiting cellular activity during different time windows of the disease. The skin, as a superficial organ, minimizes energy loss during light propagation, thereby enhancing the therapeutic signa. Delayed wound healing is typically associated with the failure of peak inflammatory suppression and the overproduction of proinflammatory cytokines resulting from bacterial infection [177]. Zhang et al. designed a spatiotemporal modulated microneedle (MN) patch for wound healing. This MN system consisted of an inner core of cross-linked heparin (cHP core) and an outer layer of polyvinyl alcohol (PVA) loaded with vitexoporfin (VP) (VP@PVA shell). Under laser irradiation, VP generated ROS to eradicate the underlying bacteria, while PVA scavenged excess ROS and mitigated the unfavorable microenvironment that stimulated inflammatory cellular immune spikes. Subsequently, the release of cHP promoted the transition of macrophages from M1 to M2, which in turn enhanced angiogenesis and extracellular matrix deposition [178] (Fig. 13). Myofibroblasts are essential cells that mediate wound closure during the repair phase. However, their over-proliferation and inadequate transdifferentiation are significant contributors to late wound scarring. To modulate the spatiotemporal disorder of myofibroblasts, another study designed a near-infrared (NIR)-responsive multifunctional artificial skin (NIR-mFAS). This system was based on chitosan and silk fibroin as the primary carriers, encapsulating epidermal growth factor (EGF) and polydopamine (pDA) nanoparticles that carried BMP4 along with the Wnt agonist CHIR99021 (pDA-NPs). NIR irradiation of the wound on day five (time) facilitated the release of BMP4 to reduce myofibroblast proliferation (space), while simultaneously releasing CHIR99021 to accelerate the transformation of myofibroblasts into cells resembling dermal papillae, thus reducing scar formation and promoting the regeneration of hair follicles and sebaceous glands [179] (Fig. 14).

**Fig. 15:**
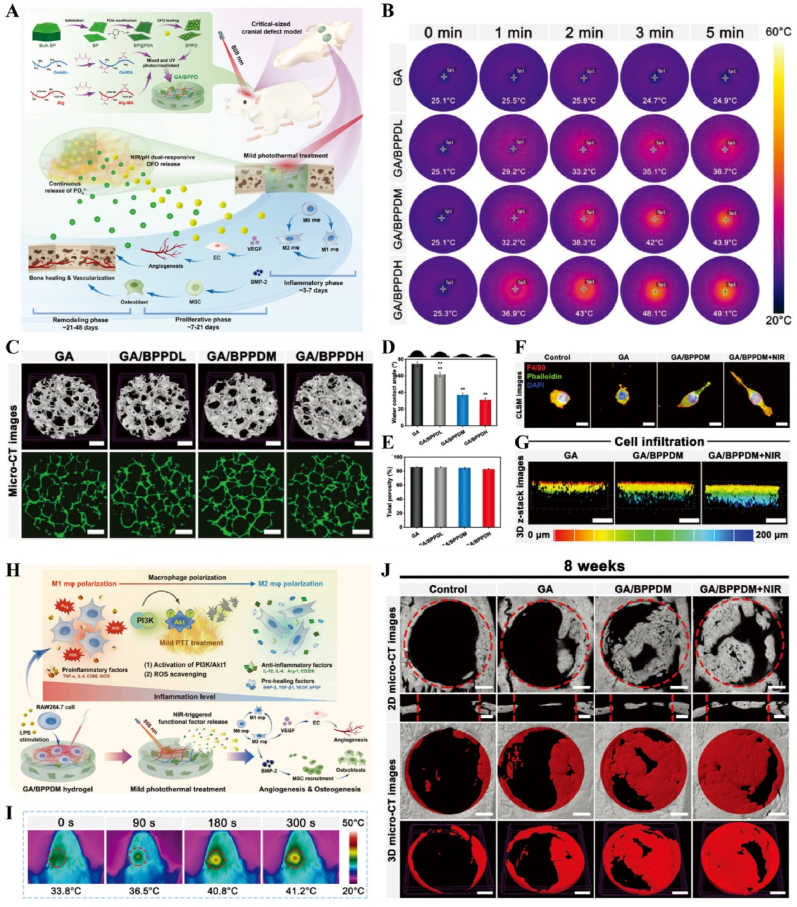
**Light-responsive biomaterials for spatiotemporal modulation in calvarial defect.** A) Schematic diagram of the production and application of GA/BPPD. B) Real-time infrared thermal images. C) Micro-CT reconstruction of the GA/BPPD hydrogel. D) water contact angle of the GA/BPPD hydrogel. E) Porosity of the GA/BPPD hydrogel. F) Immunofluorescence staining of F4/80 in RAW264.7 cells after different treatments. G) 3D images of RAW264.7 cells penetration depth. H) Schematic diagram of the potential mechanism of the GA/BPPDM hydrogel. I) Real-time infrared thermal images of rats implanted with GA/BPPD hydrogel. J) Micro-CT reconstruction of the calvarial defects [180]. Reproduced with permission. Copyright 2024, Wiley.

**Fig. 16:**
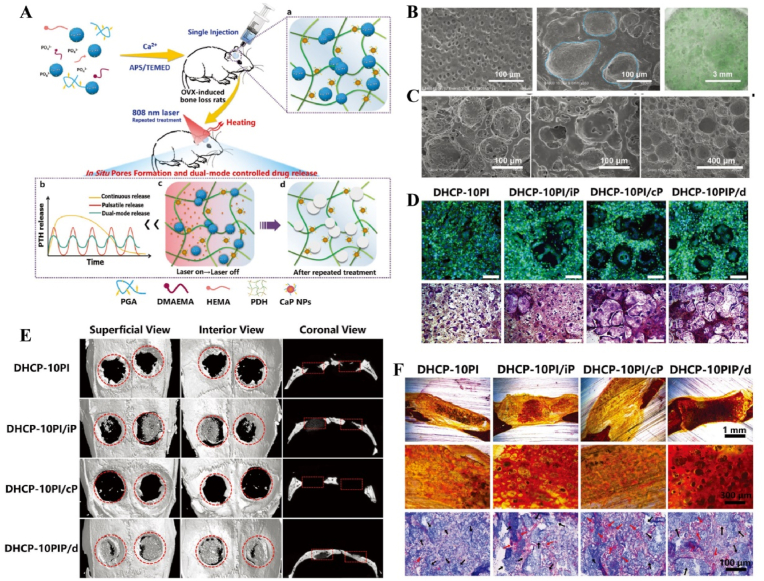
**Light-responsive biomaterials for spatiotemporal modulation in osteoporotic bone defects.** A) Schematic diagram of the application of DHCP-PIP. B) SEM and fluorescence microscopy image of DHCP hydrogel. C) SEM images of DHCP-10PI hydrogels treated with NIR irradiation, heater and after the PI MSs were completely degraded with NIR irradiation respectively. D) Fluorescence and TRAP staining images of osteoclasts. E) Micro-CT reconstruction of the calvarial defects. F) Van Gieson's staining and TRAP staining of skull [183]. Reproduced with permission. Copyright 2021, Wiley.

Light-responsive biomaterials exhibit high thermal conversion efficiency, enabling localized heating while preserving the normal temperature of surrounding tissues. A study developed a photothermal hydrogel that utilized NIR light to create a favorable immune microenvironment during the inflammatory stage and subsequently coordinates angiogenesis and osteogenesis. This hydrogel platform, known as GA/BPPD, was composed of gelatin methacryloyl (GelMA) and sodium alginate methacryloyl (AlgMA) as the structural base, and was loaded with nanosheets (BPPD) that contain polydopamine (PDA), deferiprone (DFO), and black phosphorus (BP). Under NIR irradiation, the hydrogel inhibited early immune inflammation (days 3–7), promoted osteogenic differentiation and angiogenesis (days 7–21), and contributed to late-stage bone remodeling (beyond 21 days) [180] (Fig. 15). Parathyroid hormone (PTH) is a peptide hormone that has been approved for clinical use by the US Food and Drug Administration [181]. However, in bone defect repair, local pulsed release of PTH exhibits a limited activation effect on osteoclasts, which can readily lead to a mismatch between the rate of material degradation and the pace of new bone regeneration [182]. One study utilized thermosensitive polymers, poly(N-acryloylglycinamide-acrylamide copolymer) (PNAm) and indocyanine green (ICG), to load PTH onto microspheres, forming PIP MS. PTH is released on demand *via* NIR triggering, coordinating the enhanced activities of osteoblasts and osteoclasts to align material degradation rates with bone regeneration needs in osteoporosis [183] (Fig. 16).

**Fig. 17:**
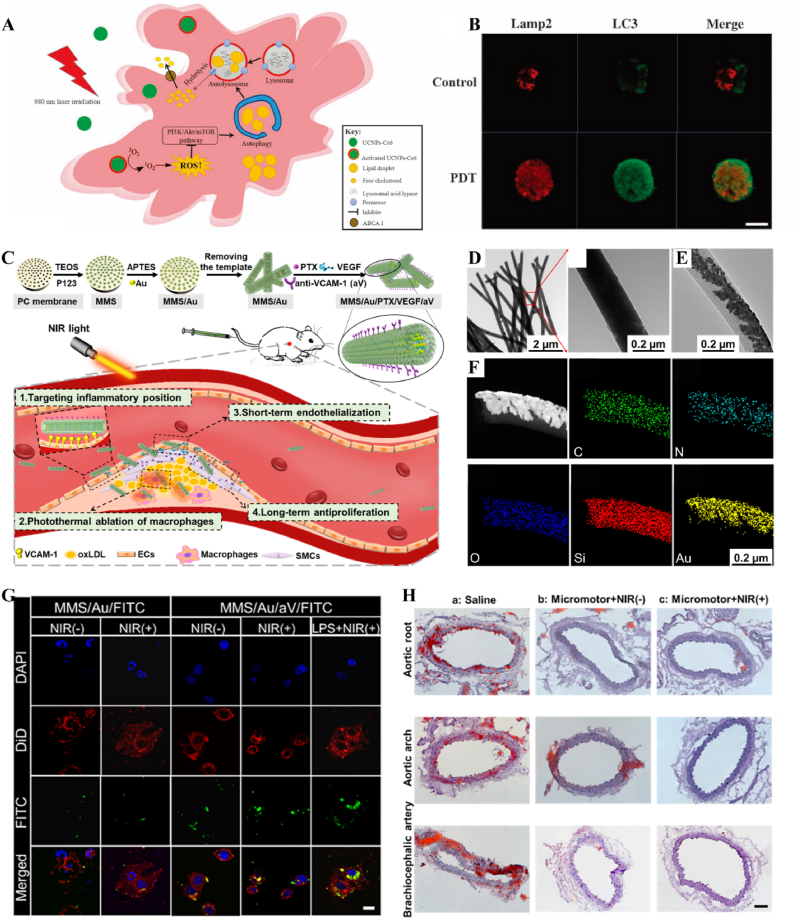
**Light-responsive biomaterials for spatiotemporal modulation in atherosclerosis.** A) Schematic diagram of UCNPs-Ce6-mediated PDT and the mechanism of autophagy. B) Confocal image of co-localization of LC3 and Lamp2 after PDT irradiation. C) Schematic diagram of MMS/Au/PTX/VEGF/aV preparation and their application in the treatment of atherosclerosis. D) TEM images of MMS. E) TEM images of MMS/Au. F) EDC mapping images of MMS/Au. G) Confocal microscopy images of HUVECs incubated with MMS/Au/FITC or MMS/Au/aV/FITC with/without the NIR laser. H) ORO staining frozen sections of aortic [185] Reproduced with permission. Copyright 2017, Springer Nature. [189]. Reproduced with permission. Copyright 2021, American Chemical Society.

In atherosclerosis, foam cells internalize LDL, a primary contributor to local hyperinflammation [184]. The traditional photosensitizer chlorin e6 (Ce6) has limited penetration depth and weak photosensitivity. By coupling Ce6 with silica nanoparticles, UCNPs-Ce6 complexes were formed. Under NIR light stimulation, which offered greater tissue penetration, the complex generated ROS to promote autophagy and cholesterol efflux in foam cells [185] (Fig. 17A and B). In recent years, light-responsive nanomotors have developed rapidly. By converting light and heat into mechanical motion at the nanoscale or microscale, these nanomotors offer unique advantages for applications in nanomedicine [[186], [187], [188]]. Li et al. developed a near-infrared (NIR)-driven multifunctional tubular micro-motor (MMS/Au/PTX/VEGF), consisting of mesoporous-macroporous silica (MMS) and gold nanoparticles (Au NPs). The larger-sized vascular endothelial growth factor (VEGF) and anti-vascular cell adhesion molecule-1 polyclonal antibody (aV) were loaded onto the surface of the tubular structure, while the smaller-sized paclitaxel (PTX) was incorporated within the mesoporous structure of the MMS. Under the influence of aV, the MMS/Au/PTX/VEGF micro-motor was targeted and enriched at the disease site. Following NIR irradiation, Au NPs activated the micro-motors, inducing thermo-swimming that allowed them to penetrate damaged blood vessels and specifically ablate foam cells, thereby treating coronary atherosclerosis [189] (Fig. 17C–H, Table 2).

**Table 2:** Light-responsive biomaterials for spatiotemporal modulation in tissue repair.

Biomaterial design	Light parameters	Application	Effects	Outcomes	Ref
Microneedle system composed of a core of cHP and an outer layer of VP@PVA shell	690 nm 25 mW cm^−2^ 10min	Wound healing	Stimulated VP and released large amounts of ROS	PF-MNs removed multi-drug resistant bacterial biofilm in early stage, regulated immune microenvironment in middle stage, and promoted scarless wound repair in late stage	[178]
Chitosan and silk fibroin as the main carriers encapsulating EGF and PDA-NPs carrying BMP4 and CHIR99021	1064 nm 1.0 W cm^−2^ 5min	Wound healing	Stimulation of PDA-NPs to release BMP4 and CHIR99021	NIR-mFAS accelerated re-epithelialization and neovascularization, promoted hair follicle regeneration and reduced scar formation	[179]
Composed of GelMA and AlgMA loaded BPPD nanosheets	808 nm 1.0 W cm^−2^ 5 min	Bone defects	Rapid released DFO and slow released PO_4_^3-^	GA/BPPD inhibited early inflammation, promoted osteogenic differentiation and angiogenesis, as well as being involved in late bone remodeling	[180]
PNAm, ICG and PTH were assembled as PNAm-ICG-PTH and loaded onto MS	808 nm 2 W cm^−2^ 45 s	Bone defects	Required release of PTH	PIP MS enhanced both osteoblast and osteoclast activity *in vitro* and *in vivo* and successfully repaired cranial defects	[183]
Ce6 combined with silica nanoparticles	980 nm 1.0 W cm^−2^ 60 s	THP-1 derived foam cells	Released ROS	Generated ROS to induce autophagy and cholesterol efflux in foam cells	[185]
Tubular micromotor formed with MMS and Au NPs as bodies loaded with VEGF, aV and PTX	808 nm 2.5 W cm^−2^ 10 min	Atherosclerosis	Penetrated into the damaged vessel and released of VEGF and PTX	Micromotor heated movement penetrated damaged blood vessels and ablated foam cells to treat coronary atherosclerosis	[189]

#### 4.1.3 Magnetism-responsive materials

**Fig. 18:**
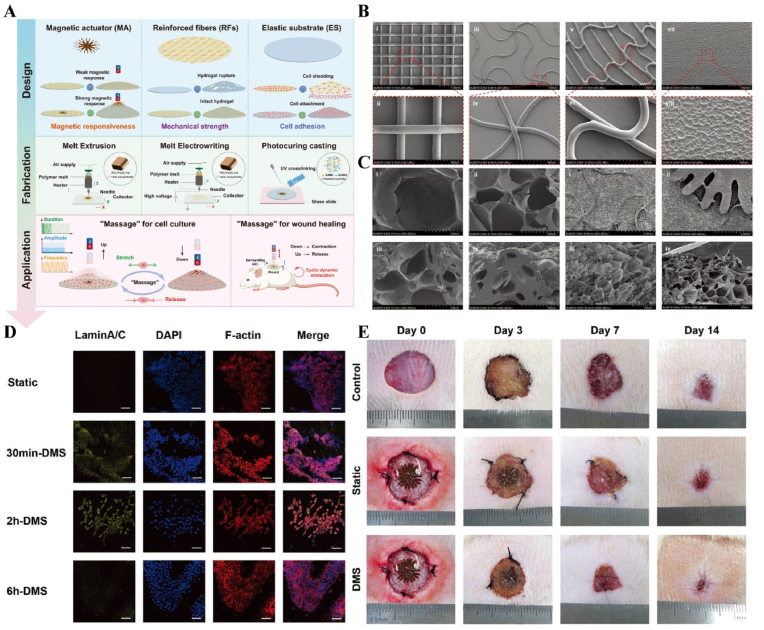
**Magnetism-responsive biomaterials for spatiotemporal modulation in wound healing.** A) Schematic diagram of MMM preparation and their application in wound healing. B) SEM images of different magnetic fibers. C) SEM images of hydrogels and MMM. D) Immunofluorescence staining of Lamin A/C with different treatments. E) Wound healing progress in different groups after surgery [194]. Reproduced with permission. Copyright 2024, Wiley.

**Fig. 19:**
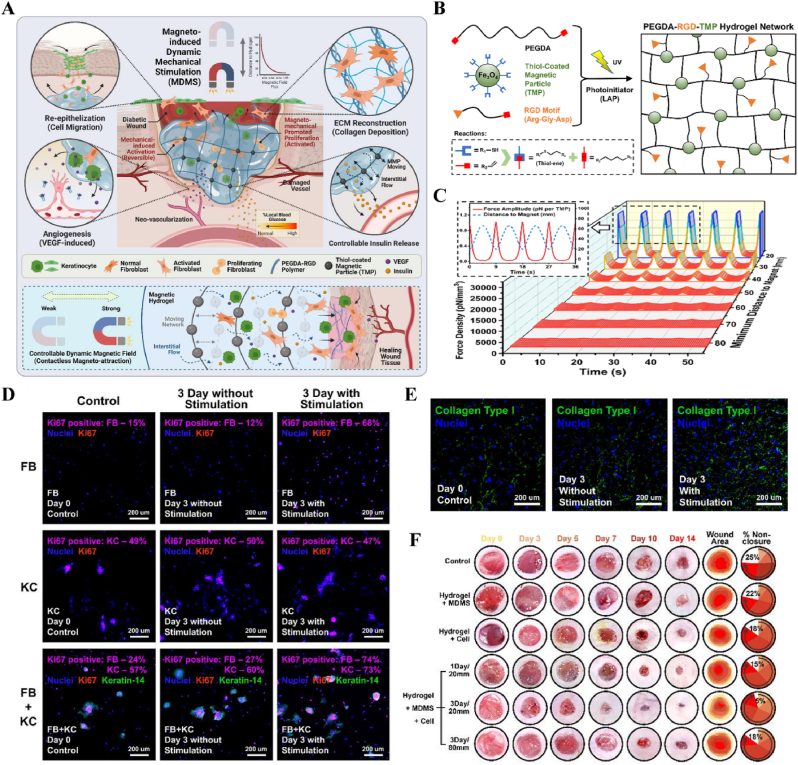
**Magnetism-responsive biomaterials for spatiotemporal modulation in diabetic wound healing.** A) Schematic diagram of PEGDA-RGD-TMP hydrogel application in diabetic wound healing and B) preparation. C) Profile of magnetic force applied to the PEGDA-RGD-TMP hydrogel. D) Multiphysics modeling of insulin concentration and distribution in hydrogel under MDMS. E) Immunofluorescence images of Ki67 and F) collagen type Ⅰ expression. G) Wound healing progress in different groups after surgery [195]. Reproduced with permission. Copyright 2023, Wiley.

In the field of spatiotemporal modulation research, magnetism-responsive materials present innovative strategies for disease treatment [190,191]. By incorporating MNPs into the material, magnetism-responsive materials can specifically optimize the microenvironment at various stages of the disease, facilitating recovery from the spatial and temporal disorders associated with the condition. The mechanical tension surrounding myofibroblasts is diminished, resulting in a reduced capacity for ECM remodeling and delayed wound closure [192,193]. One study employed magnetically responsive biomaterials (MMM) to artificially apply mechanical stimuli to the wound during the healing process, thereby accelerating wound healing. The material was composed of a soft elastic substrate (ES), reinforced fibers (RFs), and Fe_3_O_4_ NPs. During the healing process (time), the platform regulated the magnetic force and the force/deformation frequency of the stent to expose cells and tissues to varying stimuli. This promoted the expression of myofibroblast alpha-smooth muscle actin (α-SMA) and keratin 14 (K14) in keratinocytes (space), facilitated wound contraction and re-epithelialization, and consequently accelerated wound healing [194] (Fig. 18). High blood sugar can seriously impair the vitality and function of myofibroblasts and keratinocytes. Another study developed a poly (ethylene glycol) diacrylate (PEGDA)-based hydrogel (MDMS) containing a cell adhesion RGD peptide and a thiol-coated magnetic particle (TMP). MDMS was utilized to load dermal fibroblasts and epidermal keratinocytes. On the one hand, it enhanced the microenvironment by lowering glucose concentration at the wound site through insulin release, thereby mobilizing endogenous dermal fibroblasts and epidermal keratinocytes to restore function. On the other hand, it employed mechanical stimulation transmitted *via* magnetic effects to recruit exogenous dermal fibroblasts and epidermal keratinocytes into the wound repair process. Additionally, single-cell sequencing results showed that mechanical stimulation induced by magnetic forces preferentially activates fibroblasts, which integrate and transduce mechanical stimuli to regulate keratinocyte activity [195] (Fig. 19).

**Fig. 20:**
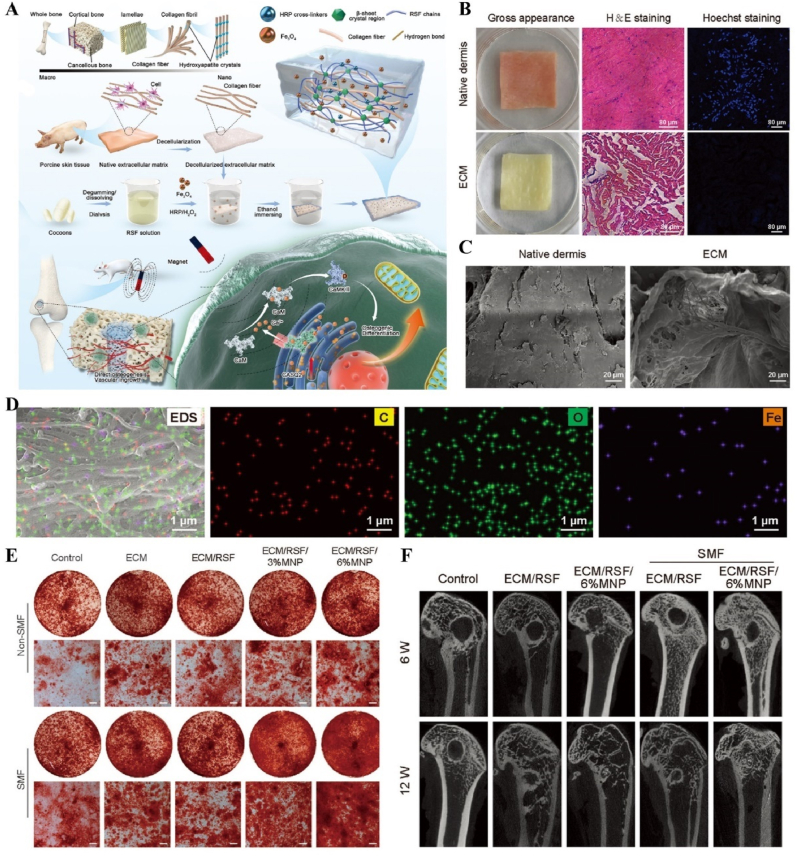
**Magnetism-responsive biomaterials for spatiotemporal modulation in bone defects.** A) Schematic diagram of ECM/RSF/MNP preparation and their application in bone defects. B) H&E staining and Hoechst staining of ECM before and after the decellularization. C) SEM of ECM. D) EDC mapping images of ECM/RSF/6 %MNP scaffold. E) ARS staining on day 21. F) Micro-CT reconstruction of the femur [198]. Reproduced with permission. Copyright 2023, Wiley.

**Fig. 21:**
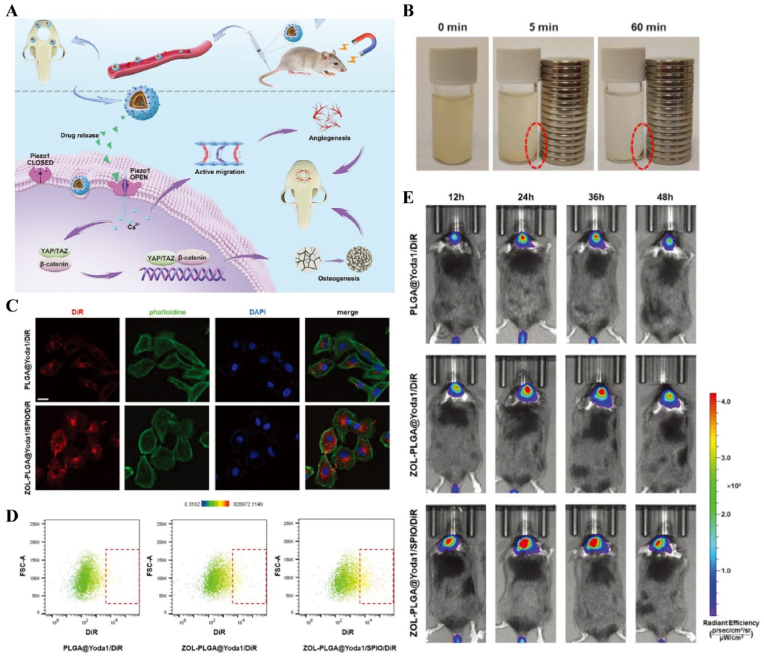
**Magnetism-responsive biomaterials for spatiotemporal modulation in osteoporotic bone defects.** A) Schematic diagram of ZOL-PLGA@Yoda1/SPIO NPs preparation and their application in osteoporotic bone defects. B) The magnetic response of ZOL-PLGA@Yoda1/SPIO NPs. C) Immunofluorescence images of HUVECs incubated with NPs. D) Flow cytometric analysis of BMSCs incubated with NPs. E) Fluorescence images of the skull after injection with DiR-labeled NPs [199]. Reproduced with permission. Copyright 2024, Wiley.

The skeletal system continuously responds to mechanical stimuli [196]. Osteoblasts participate in the bone formation process by receiving mechanical signals from stress, strain, fluid flow, and other factors, playing a crucial role in the regulation of bone homeostasis and remodeling [197]. Therefore, the mechanical properties induced by the magnetic field may guide osteoblast activity, as well as the alignment and migration of these cells. A study developed a decellularized extracellular matrix (ECM)/regenerated silk fibroin (RSF) scaffold (ECM/RSF/MNP) incorporating Fe_3_O_4_ NPs. MNPs in this biomimetic scaffold respond to an external static magnetic field (SMF), inducing mechanical stimulation that effectively promoted cell migration, osteogenic differentiation, angiogenesis, and new bone formation during the healing process. RNA sequencing results indicated that the osteogenic effect may be associated with changes in intracellular and extracellular Ca^2+^ levels [198] (Fig. 20). The mechanically sensitive Piezo1 channel is a non-selective Ca^2+^ channel, is recognized as a key sensor of mechanical signals. Guan et al. developed a magnetically responsive nanocarrier (ZOL-PLGA@Yoda1/SPIO) composed of a zoledronic acid (ZOL)-decorated PLGA nanoparticle, superparamagnetic iron oxide (SPIO), and the Piezo1-activating molecule Yoda1. ZOL-PLGA@Yoda1/SPIO was induced by a magnetic field to aggregate at the injury site and activated the Piezo1 channel in the bone defect area, promoting the osteogenic-angiogenic coupling and achieving early bone reconstruction during bone defect healing [199] (Fig. 21).

**Fig. 22:**
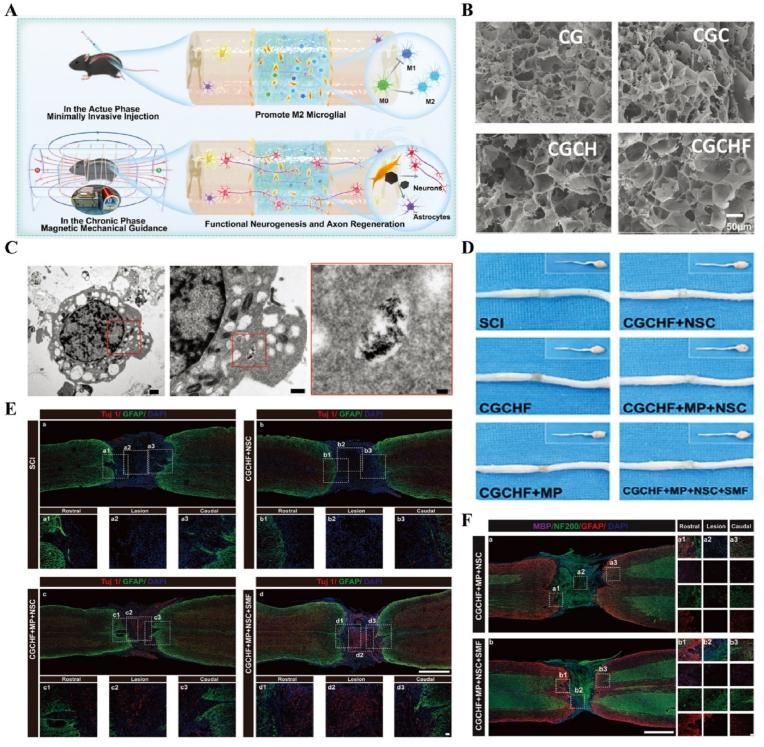
**Magnetism-responsive biomaterials for spatiotemporal modulation in SCI.** A) Schematic diagram of CGCHF preparation and their application in SCI. B) SEM images of CG, CGC, CGCH and CGCHF. C) TEM images of nanoparticles in NSCs differentiated cells. D) The morphology of the tissue at the SCI defect site with difference groups. E) Immunofluorescence images of Tuj 1 and GFAP. F) Immunofluorescence images of MBP, NF200, and GFAP [203]. Reproduced with permission. Copyright 2024, Wiley.

Regulating inflammatory cell infiltration and directing neural stem cells (NSCs) growth are two major challenges that urgently require attention in the field of SCI repair [[200], [201], [202]]. To improve inflammatory infiltration in the early stage and the disorder of directional differentiation of neural stem cells in the middle and late stages, Zhang et al. developed a nano-hydrogel containing DMSA@Fe_3_O_4_ and methylprednisolone (MP). In the acute phase, this system released MP to inhibit inflammatory infiltration. In the chronic phase, DMSA@Fe_3_O_4_ in the SMF generated mechanical stimulation to guide the proliferation and directional differentiation of NSCs into functional neurons, thereby treating SCI [203] (Fig. 22, Table 3).

**Table 3:** Magnetism-responsive biomaterials for spatiotemporal modulation in tissue repair.

Biomaterial design	Magnetism parameters	Application	Effects	Outcomes	Ref
Composed of ES, RF and Fe_3_O_4_ NPs	1 Hz 6 % 30 min	Wound healing	Adjustment of the magnetic force and force/deformation frequency of the holder to produce mechanical stimulation	MMM promoted the expression of *α*-SMA and keratinocyte K14 in myofibroblasts to promote wound contraction and re-epithelialization, which in turn accelerated wound healing	[194]
MDMS consisted of a PEGDA hydrogel containing RGD and TMP	0.1 Hz 1T with periodic displacement	Diabetic wound healing	Release of insulin reduced glucose concentration at the wound site and mechanically stimulated exogenous dermal fibroblasts and epidermal keratinocytes	MDMS promoted angiogenesis, improved paracrine secretion from keratinocytes, and on-demand release of insulin for spatiotemporal regulation.	[195]
Composed of decellularized ECM/RSF containing Fe_3_O_4_ NPs	6 % 120–130 mT 150 × 150 × 10 mm^3^	Bone defects	Mechanical stimulation occurred in response to SMF that evoked ECM/RSF/MNP	ECM/RSF/MNP scaffolds promoted cell migration, osteogenic differentiation, endothelial cell neogenesis and new bone formation	[198]
Composed of ZOL@PLGA NPs, SPIO and Yoda1	Round magnets (maximum strength = 6.6 G)	Osteoporosis bone defects	Assembled at the injury and activated Piezo1 channels	ZOL-PLGA@Yoda1/SPIO promoted osteogenic-angiogenic coupling for bone reconstruction in the early stage of bone defect healing	[199]
Composed of DMSA@Fe_3_O_4_ NPs and MP	-2T to +2T 2 h	Spinal cord injury	Released MP and mechanical stimulation occurred in response to SMF	CGCHF inhibited M1 inflammatory infiltration and directed NSCs proliferation and directed differentiation to differentiate into functional neurons	[203]

### 4.2 Chemical factors responsive materials for spatiotemporal modulation

The nature of the response of chemically responsive materials stems from biochemical processes driven by local microenvironmental changes and cellular secretory activities in the body, including the degradation of specific components or the formation of new structures. Thus, chemically responsive materials respond to objects that are responsive to biological factors in the body and play a role in regulating the microenvironment to promote tissue regeneration and repair processes. The advantage of chemically responsive materials relies in their ability to automatically detect changes in the microenvironment of the implant site and to spontaneously perform corresponding functions as needed. For example, in a diabetic wound, the abnormal biological factor pH rises to 8–9, and a responsive biomaterial developed based on the change in biological factors pH can spontaneously restore the abnormal pH, thus enhancing diabetic wound care and healing [150]. However, these materials are often disposable and irreversible. In the following sections, we will discuss the application of chemically responsive materials in disease-related modulation, focusing on the perspectives of pH and redox conditions.

#### 4.2.1 PH-responsive materials

**Fig. 23:**
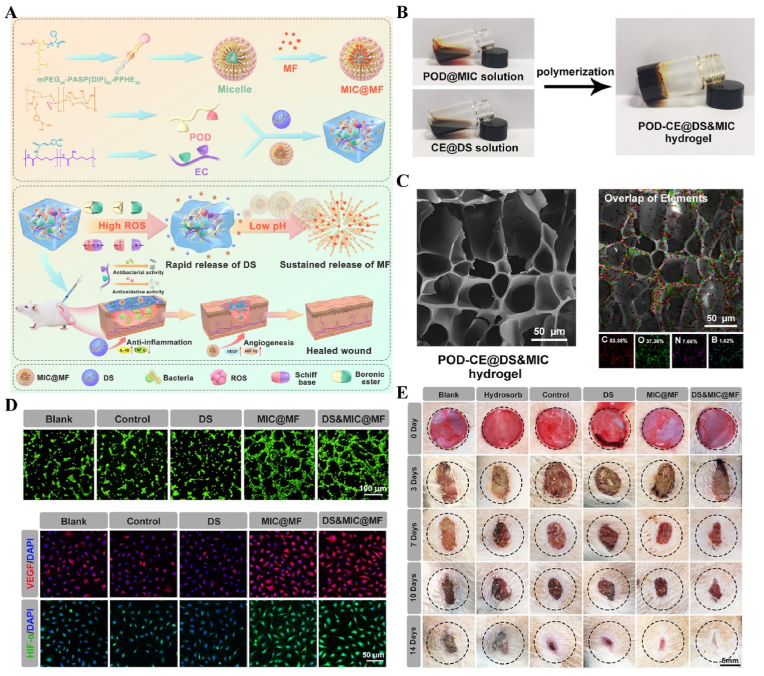
**PH-responsive biomaterials for spatiotemporal modulation in wound healing. A)** Schematic diagram of DS&MIC@MF preparation and their application in wound healing. B) Images of the hydrogel formation. C) SEM and EDC mapping images of hydrogels. D) Images of tube formation and immunofluorescence of VEGF and HIF1-α for HUVECs. E) Wound healing progress in different groups after surgery [205]. Reproduced with permission. Copyright 2022, Elsevier.

**Fig. 24:**
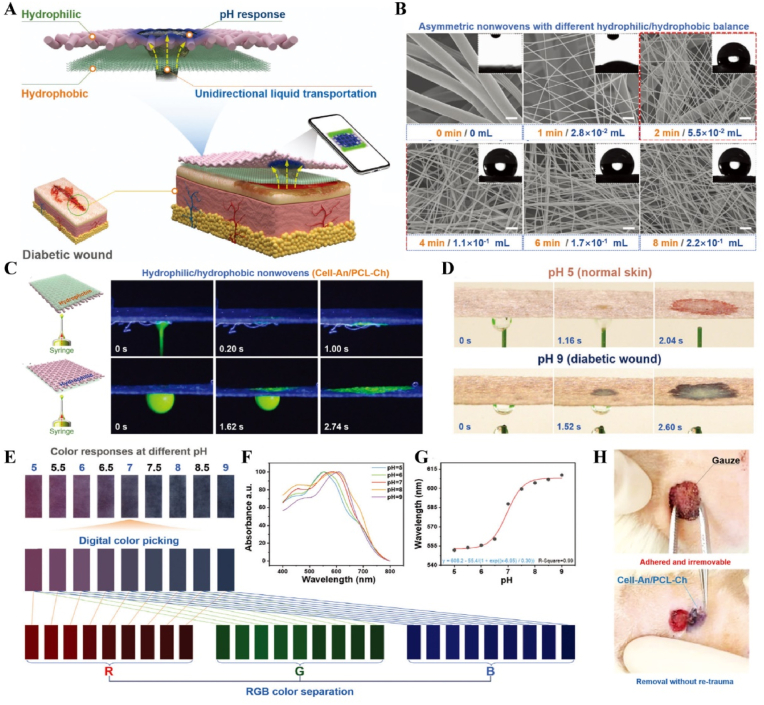
**PH-responsive biomaterials for spatiotemporal modulation in diabetic wound healing.** A) Schematic diagram of the wound dressing application in diabetic wound healing. B) SEM images of wound dressing. C) Images of the hydrophilic and hydrophobic surfaces of Cell-An/PCL-Ch. D) The color of Cell-An/PCL-Ch nonwovens at different pH values. E) Color variations of Cell-An/PCL-Ch, digital colors and the related trichromatic colors. F) UV–vis spectra, and G) the related *λ*_max_-pH dependence of Cell-An/PCL-Ch nonwovens at different pH values. H) Wound dressing removal for Cell-An/PCL-Ch [206]. Reproduced with permission. Copyright 2024, Wiley. (For interpretation of the references to color in this figure legend, the reader is referred to the Web version of this article.)

Changes in disease states are often accompanied by alterations in local pH, creating opportunities for the use of pH-responsive materials in disease treatment [204]. The advantage of pH-responsive materials lies in their ability to operate without the need for human intervention in regulating the timing of biological responses. When these materials detect changes in the surrounding pH, they can spontaneously facilitate precise drug release or regulate biological responses within specific temporal and spatial contexts. Generally, the pH of normal skin is about 4–6, while the pH of a broken diabetic wound rises to 8–9 [150]. Wu et al. developed a multifunctional hydrogel (DS&MIC@MF) composed of caffeic acid-grafted ε-polylysine (CE) and phenylboronic acid-grafted oxidized dextran (POD). The hydrogel was formed by cross-linking CE and POD *via* dynamic schiff base and borate ester bonds, and contained MIC@mangiferin (MF) and diclofenac sodium (DS). When the pH and ROS levels at the wound site change (time), the schiff base and borate ester bonds in DS&MIC@MF break, resulting in the rapid release of the CE polymer, which possesses antibacterial and antioxidant properties, as well as DS, which has anti-inflammatory activity (space), and continuously releasing MF with anti-apoptotic activity and the ability to promote angiogenesis. This material was highly compatible with the programmed healing process of infected wounds, specifically regulating spatiotemporal disorders and providing a simple yet effective strategy for the repair of chronic diabetic wounds [205] (Fig. 23). Chronic wound non-healing, caused by excessive inflammatory exudate and aberrant pH, is a common diabetic complication. This condition can result in the untimely replacement of standard gauze dressings and excessive exudate. A study utilized pH as a real-time detection indicator to develop a pH-responsive cellulose-based Janus dressing with unidirectional liquid drainage capability. This dressing is designed to monitor and prompt dressing changes, thereby accelerating the healing of diabetic wounds. The dressing (Cell-An/PCL-Ch) consists of a cellulose-anthocyanin hydrophilic layer (Cell-An) and a polycaprolactone-chlorhexidine hydrophobic layer (PCL-Ch). When applied to the wound, exudate permeates unidirectionally from the hydrophobic layer to the hydrophilic layer. The anthocyanin in the hydrophilic layer continuously monitors changes in the pH of the wound site, enhancing care for diabetic wounds [206] (Fig. 24).

**Fig. 25:**
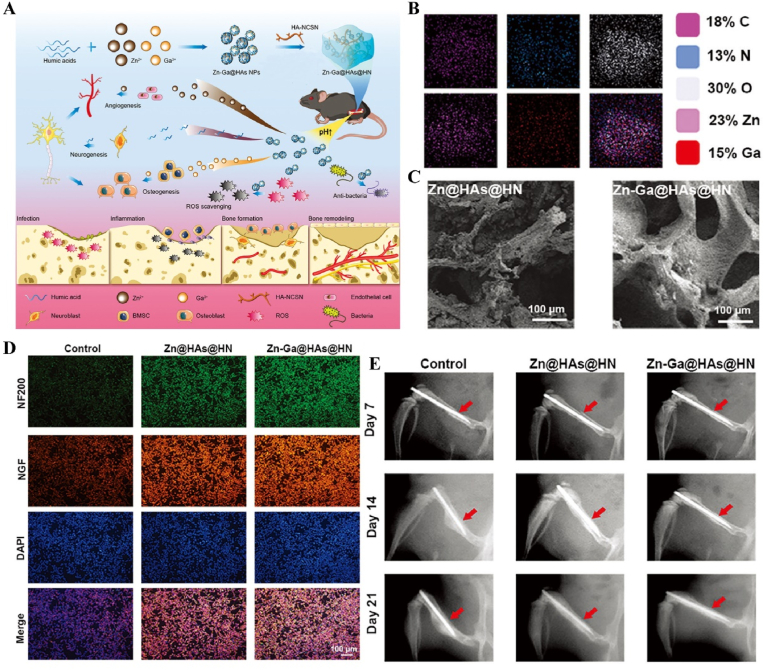
**PH-responsive biomaterials for spatiotemporal modulation in fracture healing.** A) Schematic diagram of the Zn-Ga@HAs@HN hydrogel preparation and their application in fracture healing. B) EDC mapping of Zn-Ga@HAs NPs. C)SEM images of hydrogel. D) Immunofluorescence staining of NF200 and NGF in RSC96 cells. E) X-ray images of fracture healing [207]. Reproduced with permission. Copyright 2024, Wiley.

Similar to wound healing, the physiological microenvironment at the fracture site is weakly acidic in the presence of early inflammation, vascular damage and bacterial infection. As the inflammation subsides and new blood vessels form, the pH gradually rises. Zha et al. designed a zinc-gallium humic acid (HAs) nanohybrid (Zn-Ga@HAs@HN) composed of pH-responsive Zn-Ga@HAs NPs and HA-NCSN. In the late stage of fracture healing, the wound environment becomes slightly alkaline, causing HAs to dissociate and release Zn^2+^ and Ga^3+^. The Zn-Ga@HAs@HN nanohybrid synergistically regulated angiogenesis, osteogenesis, and neurogenesis through the responsive release of Zn^2+^, Ga^3+^, and HAs, thereby promoting the spatiotemporal modulation of healing in infected fractures [207] (Fig. 25).

**Fig. 26:**
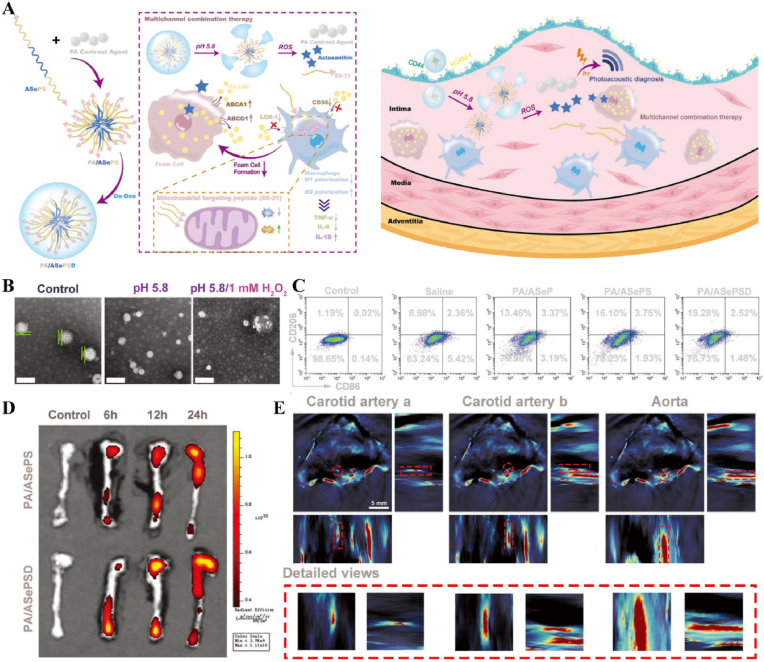
**PH-responsive biomaterials for spatiotemporal modulation in atherosclerosis.** A) Schematic diagram of the PA/ASePSD preparation and their application in atherosclerosis. B) TEM images of NPs. C) Flow cytometry analysis of macrophage polarization in difference NPs. D) Ex vivo images of aortas of ApoE^−/−^ mice treated with difference Cy5.5-labeled NPs. E) Distribution of photoacoustic signals in the aorta and carotid artery [209]. Reproduced with permission. Copyright 2023, Wiley.

The acidic microenvironment resulting from the enhanced glycolytic activity of macrophages in atherosclerosis presents a therapeutic target for inhibiting the inflammatory response and reducing cholesterol accumulation [208]. Therefore, a pH-responsive PA/ASePSD nanoparticle platform was designed to modulate M1 macrophage polarization and impaired lipid metabolism. The platform formed a shell encapsulating hydrophobically polymerized PMeTPP-MBT *via* cross-linking with oxidized dextran (ox-Dex) and SS-31 peptide. Due to the action of ox-Dex, PA/ASePSD was highly enriched at the site of endothelial injury. The acidic microenvironment triggered the cleavage of Schiff base bonds, releasing astaxanthin, SS-31 peptide and PMeTPP-MBT. Astaxanthin and SS-31 peptide jointly inhibited the peak of macrophage inflammation. These findings demonstrated that the PA/ASePSD nanoplatform ingeniously employed a combined treatment of SS-31 peptide and astaxanthin, along with photoacoustic imaging, to achieve real-time diagnosis of atherosclerosis and enable temporal control of plaques [209] (Fig. 26).

**Fig. 27:**
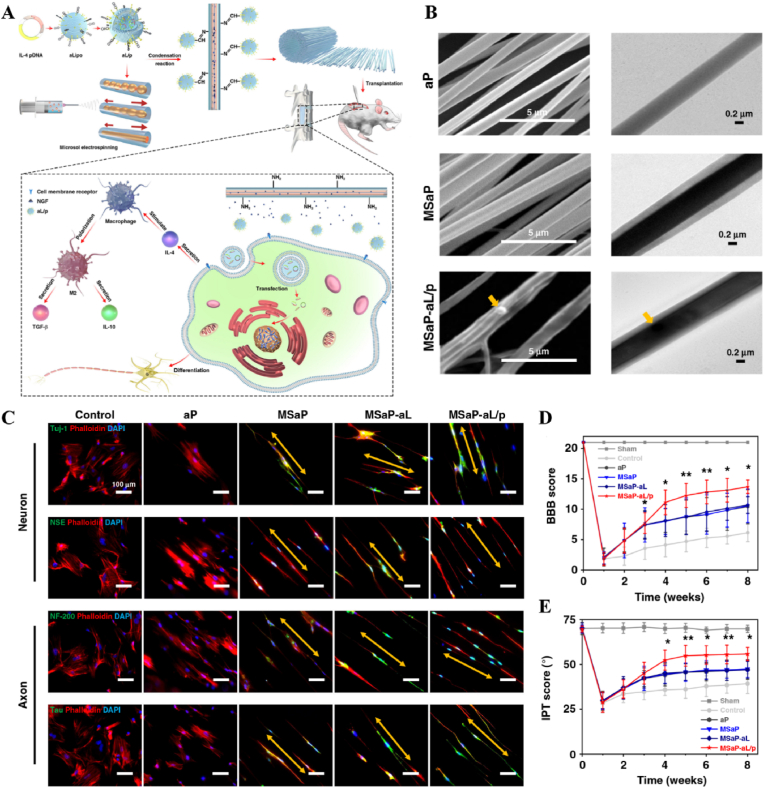
**PH-responsive biomaterials for spatiotemporal modulation in SCI.** A) Schematic diagram of the MSaP-aL/p fiber scaffold preparation and their application in SCI. B) SEM images of fiber scaffolds. C) Immunofluorescence staining of Tuj-1, NSE, NF-200, and Tau on groups. D) BBB score in rats. E) IPT score in rats [213]. Reproduced with permission. Copyright 2020, Springer Nature.

After acute SCI, the damaged tissue is acidified due to ischemia, which leads to local cell dysfunction [210]. Macrophages, which peak 3 days after injury, and activated microglia, which peak 7 days after injury, further contribute to injury and scar tissue formation [211,212]. Therefore, timely control of the peak of SCI inflammation in the acute phase to create a favorable repair environment is crucial for late nerve regeneration. Xi et al. developed a directional microemulsion electrospun fiber scaffold (MSaP-aL/p) loaded with interleukin-4 (IL-4) plasmid DNA (pDNA) liposomes. This system comprised aldehyde-modified cationic liposomes that were grafted onto the surface of the directional microemulsion electrospun fiber scaffolds *via* a schiff base reaction, forming a dynamic covalent structure. In an acidic environment, the IL-4 plasmid was released, inducing M2 polarization and anti-inflammatory factor secretion in macrophages and microglia, inhibiting acute inflammation and rapidly regulating immune function, paving the way for subsequent nerve regeneration [213] (Fig. 27, Table 4). For neurodegenerative diseases, the BBB is a key factor that makes time-controlled delivery difficult to achieve. A study has developed an acid-responsive cascade-targeted nanomodulator (Res@TcMNP/ASO) for the treatment of AD. It can penetrate the BBB, precisely identify and accumulate in abnormal acidic environments, target microglia, inhibit neuroinflammation, and improve cognitive function [214].

**Table 4:** PH-responsive biomaterials for spatiotemporal modulation in tissue repair.

Biomaterial design	Response component	Application	Effects	Outcomes	Ref
Composed of CE and POD crosslinked by dynamic Schiff base and borate bonds and containing MIC@MF and DS	Schiff base and borate bonds	Diabetic wound healing	Rapid released CE and DS, slow released MF	DS&MIC@MF exerted effective anti-infective, antioxidant and anti-inflammatory effects, further promoted angiogenesis and accelerating wound repair.	[205]
Composed of a hydrophilic layer (Cell-An) and a hydrophobic layer (PCL-Ch)	pH-sensitive component (An)	Diabetic wound healing	Absorption of exudate, reflecting the pH microenvironment	Cell-An/PCL-Ch alleviated hyperglycemic exudate flooding, monitored pH changes at the wound site for better diabetic wound care	[206]
Composed of Zn-Ga@HAs NPs and HA-NCSN	Humic acids	Infected bone defects	Released Zn^2+^, Ga^3+^ and HAs	Zn-Ga@HAs@HN regulated angiogenesis, osteogenesis and neurogenesis for infected bone defect repair	[207]
Cross-linking of ox-Dex and SS-31 peptides to form a shell-encapsulated hydrophobically polymerized PMeTPP-MBT	Schiff base bonds	Atherosclerosis	Schiff base bonds were broken and astaxanthin, SS-31 peptide and PMeTPP-MBT were released	PA/ASePSD nanoplatform for real-time diagnosis of atherosclerosis and suppression of macrophage inflammatory peaks	[209]
Composed of aL/P grafted onto the surface of oriented microsol electrostatically spun fiber scaffolds	Schiff base bonds	Spine cord injury	Schiff base bonds were broken and aL/P were released	Induced M2 polarization and anti-inflammatory factor secretion in macrophages and microglia and indirectly promoted the synthesis and release of NGF	[213]

#### 4.2.2 Redox-responsive materials

**Fig. 28:**
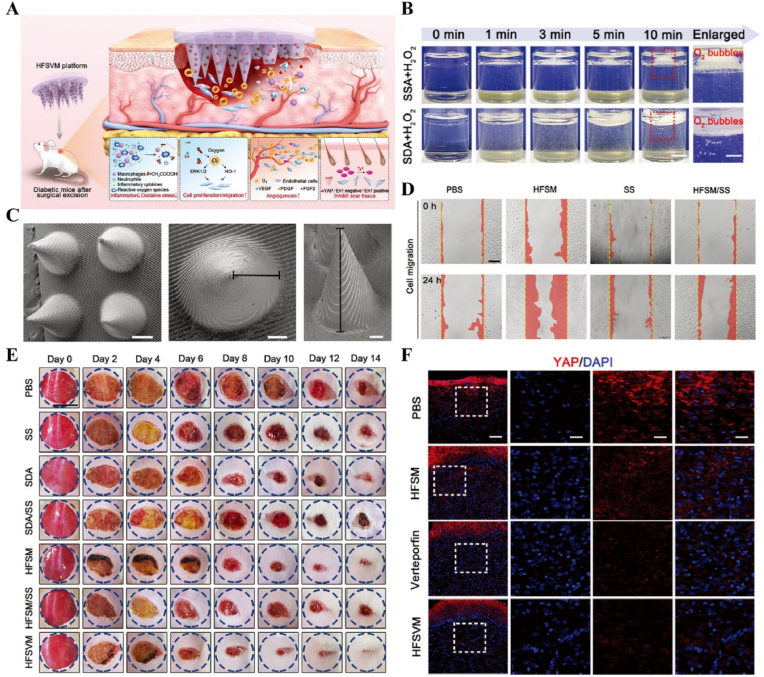
**Redox-responsive biomaterials for spatiotemporal modulation in diabetic wound healing.** A) Schematic diagram of the HFSVM platform application in diabetic wound healing. B) Images of Oxygen bubbles in the SDA-H_2_O_2_ and SSA-H_2_O_2_ system. C) SEM images of HFSVM. D) Images of HUVECs migration treated with difference groups. E) Wound healing progress in different groups after surgery. F) Immunofluorescence staining of YAP at wound [218]. Reproduced with permission. Copyright 2024, Wiley.

**Fig. 29:**
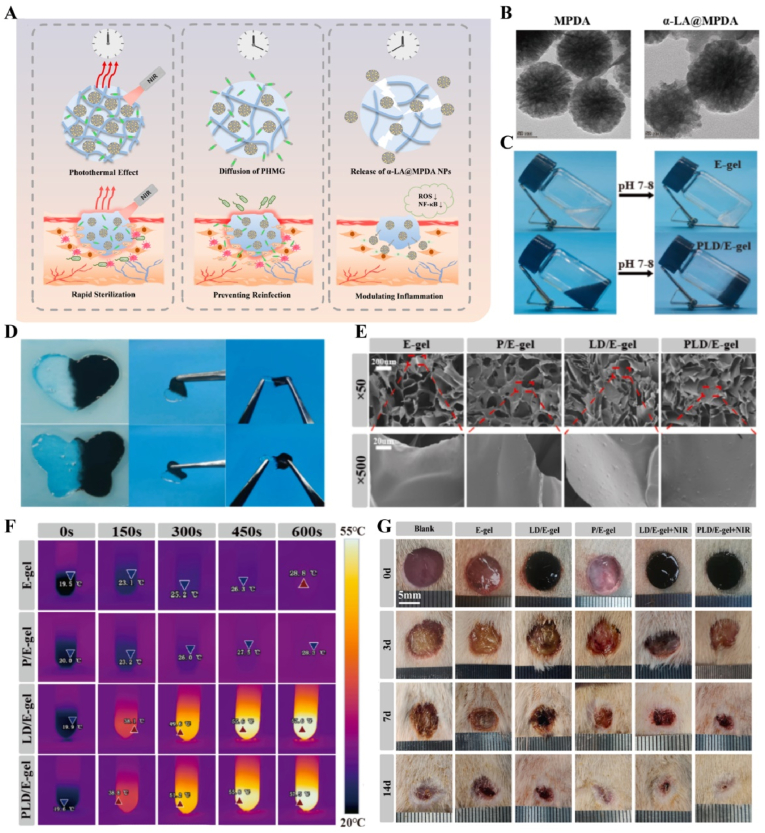
**Redox-responsive biomaterials for spatiotemporal modulation in diabetic wound healing.** A) Schematic diagram of the PLD/E-gel application in diabetic wound healing. B) Internal structure of MPDA and α-LA@MPDA NPs. Images of gel-forming C) properties, D) self-healing properties. E) SEM images of E-gel, P/E-gel, LD/E-gel and PLD/E-gel. F) Real thermal imaging photograph of E-gel, P/E-gel, LD/E-gel and PLD/E-gel. G) Wound healing progress in different groups after surgery [219]. Reproduced with permission. Copyright 2023, Elsevier.

Redox-responsive materials have attracted increasing attention in the study of spatiotemporal-controlled regulation and have emerged as a significant direction in disease treatment [215,216]. Excessive ROS poses a major obstacle in the wound healing process associated with diabetes. They can cause irreversible damage to cells and sustain macrophages in the M1 phenotype, thereby exacerbating the inflammatory response [217]. Therefore, supplementing oxygen deficiency is of great clinical significance in promoting wound healing in diabetes. A hydrogel microneedle (HFSVM) was synthesized using dopamine-functionalized sericin protein (SDA) and 4-amino-3-fluorophenylboronic acid-functionalized hyaluronic acid (HA-FPBA), with verteporfin (VP) preloaded into the structure. When SDA in the hydrogel encountered excessive ROS, it triggered the decomposition of peracetic acid to produce O_2_, alleviating wound hypoxia, inhibiting the expression of pro-inflammatory cytokines, and promoting cell migration and angiogenesis. In addition, high glucose concentrations caused the dissociation of the borate ester bond in the FPBA, releasing VP, which inhibited fibroblast proliferation and differentiation [218] (Fig. 28). ROS is a double-edged sword. The production of ROS is beneficial for killing bacteria during the inflammatory phase, but excessive ROS is not conducive to wound healing during the repair phase. Sun et al. developed a PLD/E hydrogel composed of α-lipoic acid (α-LA) @PDA nanoparticles and polyhexamethylene guanidine (PHMG). This hydrogel consumed excess ROS produced early by killing bacteria and released α-LA to exert an anti-inflammatory effect, promoting the wound to enter the repair phase [219] (Fig. 29).

**Fig. 30:**
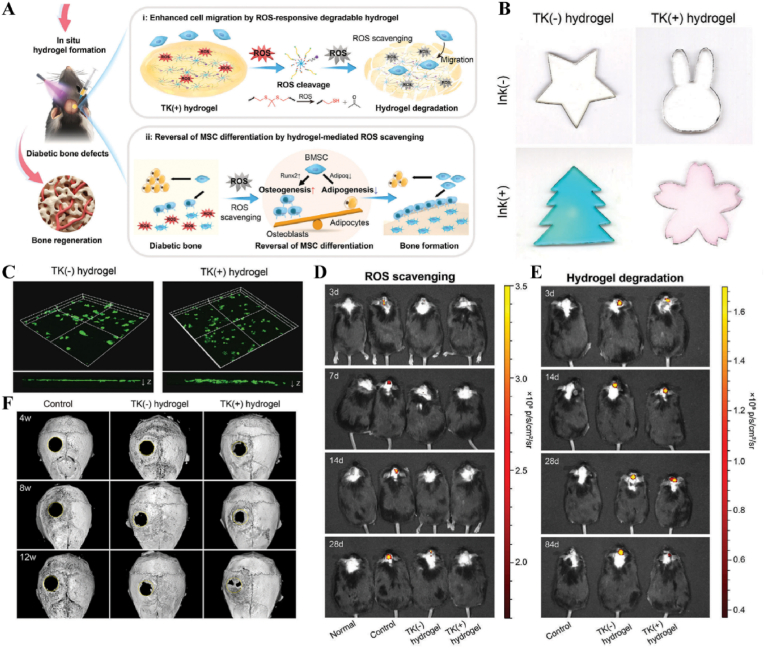
**Redox-responsive biomaterials for spatiotemporal modulation in Diabetic bone healing.** A) Schematic diagram of TK(+) hydrogels application in diabetic wound healing. B) Images of injectability and plasticity of hydrogels. C) Migration analysis of BMSCs on the hydrogels. Fluorescence images of D) ROS scavenging and E) hydrogels degradation. F) Micro-CT reconstruction of the calvarial defects [221]. Reproduced with permission. Copyright 2024, Wiley.

Hypoxia-induced excessive ROS production in the bone defect area delays bone regeneration and healing [220]. A study synthesized a hydrogel system conjugated with a ROS-cleavable thioketone (TK) linker. This hydrogel system can protect cells from oxidative stress by consuming excess ROS resulting from early hypoxia and can induce BMSCs to differentiate into adipocytes while also promoting osteogenesis through differentiation into osteoblasts [221] (Fig. 30). However, the consumption of excessive ROS through the rapid addition of O_2_ can disrupt the redox balance, adversely affecting the activity of osteoblast precursor cells and hindering bone regeneration [222]. A study combined perfluorocarbon (PFC)@PLGA/PPS NPs with catalase (CAT) and loaded them into liposomes, which were then encapsulated with GelMA hydrogels to construct CPP-L/GelMA. The system utilized PFC to generate O_2_ to alleviate hypoxia and employed CAT to scavenge excessive ROS, thereby adapting to the dynamic needs of the microenvironment in the defective area. In summary, this study addressed the challenges posed by the hypoxic microenvironment in bone defect regions, ingeniously regulating the balance between hypoxia and ROS production, thereby promoting angiogenesis, inhibiting osteoclast differentiation, and facilitating osteoblast differentiation [223].

NO is essential for preventing platelet aggregation, regulating the phenotype of smooth muscle cells and maintaining endothelial cell function [224]. However, high levels of ROS in the microenvironment severely impair the efficacy of NO-based therapies [225]. Li et al. developed a supramolecular drug scaffold in which ethyl caffeate (ECA) was initially conjugated to the surface of hydroxypropyl-β-cyclodextrin (HP-β-CD) *via* a borate ester bond. Subsequently, the nitric oxide (NO) donor isosorbide dinitrate (ISDN) was encapsulated within the cavity of HP-β-CD, resulting in the formation of the t-PBA&NO NP. The t-PBA&NO NP functions by consuming ROS and releasing ECA to alleviate endoplasmic reticulum stress. This process created a favorable environment that facilitates the subsequent release of NO from ISDN, thereby enhancing the bioavailability of NO and regulating endothelial dysfunction [226].

**Fig. 31:**
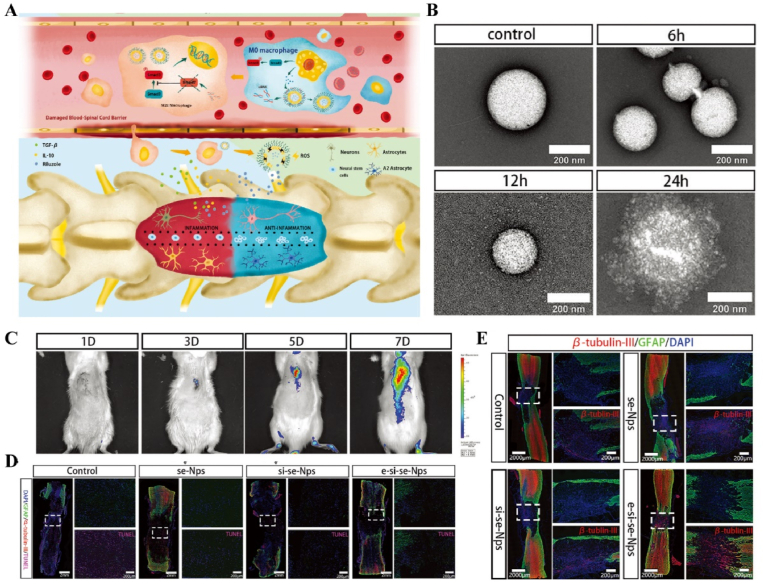
**Redox-responsive biomaterials for spatiotemporal modulation in SCI.** A) Schematic diagram of “chocolate chip cookie” application in SCI. B) TEM images of NPs. C) *In vivo* imaging of SCI rats at different time points. D) Immunofluorescence staining of neuronal apoptosis in difference groups. E) Immunofluorescence staining of neuronal regeneration and infiltration in difference groups [228]. Reproduced with permission. Copyright 2024, Wiley.

In addition to the massive production of ROS, the peak of immune inflammation and neurotoxicity that occurs 2–3 days after SCI is also a key factor affecting nerve regeneration [227]. A ROS-responsive nanoparticle was combined with artificially aged red blood cells to create the e-si-se-Nps system, which was loaded with Smad7 siRNA and carries riluzole internally. The results demonstrated that the system significantly reduced neuronal apoptosis, preserved neurological function, improved the inflammatory microenvironment following spinal cord injury, and created favorable conditions for subsequent recovery of neurological function [228] (Fig. 31). In summary, this study presented an innovative approach to regulating the spatiotemporal disorders associated with three pathological factors of spinal cord injury, thereby opening new horizons for the integration of medicine and engineering in disease treatment (Table 5).

**Table 5:** Redox-responsive biomaterials for spatiotemporal modulation in tissue repair.

Biomaterial design	Response component	Application	Effects	Outcomes	Ref
Crosslinked from SDA and HA-FPBA and loaded with VP	SDA and borate bonds in FPBAs	Diabetic wound healing	Decomposition of peroxyacetic acid to produce O2 and dissociation of borate bonds to release VP	Inhibited expression of pro-inflammatory cytokines, promoted cell migration and angiogenesis, and inhibited fibroblast proliferation and differentiation	[218]
Composed of α-LA@PDA NPs and PHMG	ROS degraded disulfide bonds	Diabetic wound healing	Released α-LA@MPDA	Consumption of early generated ROS and released α-LA exerted anti-inflammatory effects	[219]
Composed of 8-arm-TK-NP, HS-PEG-SH and RGD	ROS cleaved TK	Diabetic bone defects	Scavenged ROS and promoted the migration of BMSCs	Protected cells from oxidative stress, induced a shift in the differentiation of BMSCs toward adipocytes to osteoblasts, and promoted osteogenesis	[221]
PFC@PLGA/PPS NPs conjugated with CAT were loaded into liposomes and encapsulated with GelMA hydrogels	ROS consumed by CAT	Bone defects	Demand-based oxygenation and scavenged ROS	Scavenged ROS, generated oxygen, promoted osteoblast differentiation, inhibited osteoclast formation, and promoted neovascularization, ultimately accelerating bone regeneration	[223]
ECA covalent to the surface of HP-β-CD and ISDN loaded into the cavity of HP-β-CD	ROS cleaved the borate bond to release ECA	Implants induced damage of endothelium	Cleared ROS, released ECA and NO	Depletion of ROS, alleviation of endoplasmic reticulum stress, and modulation of endothelial dysfunction	[226]
Composed of PEI-SeSe-PCL-Se-Se-PEI NPs bound to artificially aged erythrocytes containing Smad7-siRNA and riluzole	Diselenide bonds	Spine cord injury	Promoted phagocytosis by macrophages	Targeted peripheral blood macrophages to promote macrophage conversion to an anti-inflammatory phenotype and release riluzole to protect neurological functions	[228]

### 4.3 Multi-factors joint responsive spatiotemporal modulation strategies

As described above, in the complex physiological process of tissue repair, spatiotemporal microenvironmental disturbances (e.g., abnormal inflammatory factor gradients, dysregulated extracellular matrix remodeling, etc.) are a central problem that impedes the regenerative process. Traditional single regulatory tools are often difficult to fully respond to this dynamically changing pathological microenvironment. In recent years, the rapid development of responsive biomaterials has provided new solutions to this challenge, among which physical and chemical factors responsive materials are each characterized by their own features, showing complementary regulatory advantages. Physical factors responsive materials are characterized by controllable, fixed-point, and precise effects, and different biological effects can be achieved by changing the parameters of physical factors. Ultrasound, light, and magnetism are examples of physical factors that can achieve precise spatiotemporal modulation of cellular behavior and tissue microenvironment by virtue of their programmable frequencies, intensities, and wavelengths. In wound healing, low-frequency ultrasound (1 MHz, 0.3 W/cm^2^) promoted macrophage conversion to an anti-inflammatory phenotype, whereas high-frequency ultrasound (3 MHz, 1.5 W/cm^2^) accelerated collagen remodeling through thermal effects, a frequency-dependent effect that had been used to staged modulation of the immune microenvironment in ultrasound tissue engineering [28]. However, the quantitative-effect relationship between physical factors and biological effects has not been fully elucidated, which limits the application of multiparameter co-modulation in tissue repair. In addition, most of the existing physical factors responsive materials can only achieve ranged modulation (in observable spatiotemporal disorder), making it difficult to precisely intervene against initiating factors in the localized microenvironment. Chemically responsive materials based on biological factors (pH and redox) exhibit unique spatiotemporal precision by mimicking the endogenous signaling mechanisms of biological systems, which are capable of sensing pathological microenvironmental changes in real time and triggering targeted repair behaviors. The advantages present in chemically factors responsive materials can be complemented with the disadvantages of physical factors responsive biomaterials to cope with more complex microenvironmental alterations. Recognizing the complementary properties of physical and chemical factors responsive materials, our group predeveloped a multifactor-responsive biomaterial for complex diabetic wound healing. The high glucose factor-responsive biocomponent GOx-MnO_2_ nanocontainer was introduced based on the phenomenon of early glucose augmentation and visualized to guide ultrasound-mediated angiogenesis after recovery from the hypoglycemic microenvironment, resulting in accelerated repair of diabetic wound trauma (Fig. 7) [166]. Aβ deposition and harmful inflammation are the two major spatiotemporal disorders present in the abnormal brain regions of AD. Wang et al. designed a near-infrared-II region aggregation-induced emission (AIE) nanodiagnostic and therapeutic system for precise AD treatment. By combining physical and chemical factors, this system can accurately monitor BBB penetration and the specific binding of the nanodiagnostic and therapeutic system to plaques, and activate the AD self-enhancing therapeutic process under ROS triggering [229]. This physico-chemical multifactorial diagnostic and therapeutic strategy is expected to break through the limitations of existing tissue repair techniques and provide innovative solutions for precision regenerative medicine under complex pathological conditions. In addition, both magnetic stimulation and light stimulation possess excellent remote-control capabilities, enabling precise intervention in the spatiotemporal disruption processes of diseases through a division-of-labor approach. A study developed a nano-adsorbent with dual photomagnetic stimulation response for selective removal of blood lipids. On one hand, photo construction is used to achieve lipid adsorption; on the other hand, magnetic response is utilized for rapid enrichment and separation of the adsorbent [230]. The combined use of multiple physical factors for spatiotemporal regulation of diseases represents a new strategy to overcome the limitations of single-factor tissue repair approaches. Therefore, a lot of efforts are needed to analyze the molecular mechanisms of microenvironmental dynamics during tissue repair, develop more precise responsive materials, and establish standardized clinical pathways for the spatial and temporal regulation of tissue repair.

## 5 Summary and prospects

During the progression of a disease, the unique biological characteristics and changes in the microenvironment at each stage significantly influence its trajectory. Although the mechanisms underlying the spatiotemporal changes in disease progression remain incompletely understood, the ability of intelligent responsive biomaterials to exert temporal and spatial control over diseases is evident. This paper reviews the spatiotemporal changes associated with various systemic diseases to elucidate better the spatiotemporal disorders that occur during disease progression. While intelligent responsive biomaterials are still in their infancy, they hold considerable promise as a novel approach for multi-stage and multi-type regulation of spatiotemporal changes. This review provides a comprehensive overview of current treatment strategies that utilize smart responsive materials with physical and chemical factors to regulate the spatiotemporal changes of systemic diseases. It serves as a reference for the future development of advanced biomaterials capable of modulating spatiotemporal changes in order to meet the therapeutic needs of diverse diseases. We also categorized various smart-responsive biomaterials (Table 6). Nonetheless, significant challenges remain.

1) The spatiotemporal disorders in disease are closely related to individual differences, lesion locations and microenvironmental properties. The development of responsive biomaterials needs to be centered on the disease process and deeply analyze the molecular mechanism of disease-causing phenotypes. At the same time, it is necessary to elucidate the spatiotemporal disorder triggered by individual differences, spatial heterogeneity and dynamic changes in the microenvironment. This basic research is the key to break through the bottleneck of precise regulation.
2) The therapeutic effect of physically responsive materials is highly dependent on specific device types and precisely regulated parameters, but a unified standardization system has not yet been established for research parameters in this field. This lack of standardization of key parameters not only severely limits the optimal design and clinical application of materials, but also hinders the comparability and reproducibility of research results. Future research needs to explore the following two dimensions: on the one hand, it is necessary to systematically elucidate the quantitative relationship between different physical parameters and biological effects *in vivo*; on the other hand, it is necessary to comprehensively assess the sensitivity of biomaterials to various physical parameters and their thresholds of action. Only through this kind of in-depth research from multiple perspectives can we accurately verify the regulation mechanism of disease microenvironment by physically responsive materials and provide reliable evidence of their effectiveness for clinical applications.
3) Although physical factors responsive materials have advantages such as precise on/off regulation characteristics, they still face important challenges in practical applications: on the one hand, the existing technology has limited ability to regulate high-gas content or high-density tissues, and there is a contradiction between insufficient depth of tissue penetration and accumulation of thermal effects in photodynamic therapy; on the other hand, due to the significant individual variability of spatiotemporal disorders of diseases, the current accuracy of the regulation of the parameters of physical stimuli On the other hand, due to the significant individual variability of disease spatial and temporal disorders, the accuracy of the current regulation of physical stimulation parameters can hardly meet the demand for personalized treatment. Therefore, future research should be devoted to the development of a new generation of smart response materials, not only to achieve precise intervention for different anatomical sites and disease stages, but also to break through the traditional therapeutic scope, synchronous integration of prevention, real-time monitoring and therapeutic functions. Two key issues need to be addressed: first, personalized algorithms to optimize physical parameters (e.g., stimulation time and intensity), and second, the development of dynamic response systems that can adapt to patient-specific pathological changes, thus truly realizing the paradigm shift from “standardized treatment” to “precision medicine”.
4) Although chemically responsive biomaterials can realize autonomous response based on microenvironmental changes, their clinical applications still face several key challenges. First, these materials often exhibit irreversible chemical reaction properties, resulting in a “one-time” response behavior, making it difficult to achieve long-term, dynamic and continuous regulation, which is particularly prominent in the treatment of chronic diseases. Secondly, the target specificity of the material after systemic administration is insufficient, which may trigger off-target effects in normal tissues with similar microenvironmental characteristics to the target site. More importantly, the balance between the response threshold of chemically factors responsive materials and the physiological microenvironmental homeostasis has not been clarified: an overly sensitive response may lead to premature activation or nonspecific release, whereas a sluggish response may compromise therapeutic efficacy. Therefore, future research needs to explore the relationship between the chemical composition of chemically factors responsive materials and the response threshold within the biological factors of the internal environment, focusing on the development of chemically bonded and self-feedback-regulated systems with reversible/repeatable responses to achieve a dynamic balance between therapeutic efficacy and safety. These breakthroughs will significantly enhance the application value of chemically factors responsive materials in precision medicine.
5) The spatiotemporal disorder during tissue repair is highly complex, and this dynamically changing pathological microenvironmental feature determines that it is difficult to realize precise repair by a single regulatory means. In the face of the heterogeneity of spatiotemporal characteristics at different stages of tissue repair, it is necessary to integrate the precise navigation advantages of physical factors and the environmental response characteristics of chemical factors to construct a multifactorial synergistic regulatory system with spatial and temporal resolution. Taking the strengths of physical factors and complementing the weaknesses of chemical factors can precise interventions at multiple scales of molecules, cells and tissues in response to spatiotemporal disorders. Future research needs to focus on solving key scientific problems such as multifactorial synergistic response mechanisms and providing transformative solutions for complex tissue repair through the development of novel biomaterials with spatiotemporal modulation.
6) In the development of tissue repair materials, covalent binding of multiple types is a key technology indispensable for material functionalization. Considering the stability requirements for long-term *in vivo* application of materials, it is necessary to ensure the continuity of therapeutic effects. In the process of material design and preparation, priority should be given to the selection of functionally stable chemical bonds and simple synthesis pathways, avoiding the introduction of too many complex chemical reaction steps, which not only helps to maintain the structural integrity of the material, but also effectively avoids the possible non-specific interactions and potential side effects. This balance between functionality and stability is particularly important for the development of long-term implantable materials for complex tissue repair processes.
7) Currently, physically responsive materials generally have insufficient degradation performance, especially physically responsive biomaterials, whose non-degradable or slow degradation characteristics may cause long-term complications such as chronic inflammation, foreign body reaction, and even carcinogenic risk. Therefore, future material design should take into account the principle of function-degradation-safety. While ensuring precise spatial and temporal regulation of function, the degradation kinetics of the material should be precisely regulated to match the tissue repair process; and biocompatibility should be enhanced by simplifying the material composition and optimizing the surface properties. A perfect safety evaluation system should be established in material science to provide a more reliable assessment system for clinical application.

**Table 6:** Various types of biomaterials for tissue repair spatiotemporal regulation.

Types of biomaterials	Biomaterials Composition	Factors	Response parameters	Application	Spatiotemporal modulation properties	Ref
Electrostatic spinning	Li-doped ZnO/PLLA microfibers with antioxidant 4OI coatings	US	early 1 MHz 1.5W cm2 15min late 1 MHz 0.3W cm2 1min	Infected wound healing	Early release of ROS for antibacterial and late release of antioxidants for repair	[165]
	Composed of a hydrophilic layer (Cell-An) spinning and a hydrophobic layer (PCL-Ch) spinning	pH	Reflects PH > 7.5 and PH < 6.5	Diabetic wound healing	Maintains wound wet adhesion and avoids exudate retention, pH real-time monitoring of adverse wound microenvironments	[206]
	Directed Electrostatic Spinning Fiber Scaffolds Enriched with IL-4 Plasmid	pH	Schiff base bonds	Spine cord injury	Acute phase release of IL4 plasmid rapidly modulates immune effects	[213]
Hydrogel	HA hydrogel enriched with dual nanoparticles	US	650 kHz 1W cm^2^ 1min	Diabetic wound healing	Precise release of VEGF	[166]
	GelMA/HAMA hydrogels loaded with RES@PLGA nanoparticles	US	650 kHz 1.5W cm^2^ 1min	Bone defects	Release of ultrasound-responsive nanoparticles on the first day after injury	[4]
	Sodium alginate hydrogel with Eb and hydroxyapatite	US	1.5 MHz 0.4 Mpa 10 min	Bone defects	Rapid-released of Eb for ROS scavenging and slow-released Ca^2+^ for osteogenic differentiation of BMSCs	[172]
	Chitosan and silk fibroin Hydrogel loaded with BMP4 and CHIR99021	Light	1064 nm 1.0 W cm^2^ 5min	Wound healing	NIR-mFAS rapid sustained release of EGF	[179]
	GelMA/AlgMA loaded with BPPD nanosheets	Light	808 nm 1.0 W cm^2^ 5 min	Bone defects	Rapid release of DFO by photothermal effect Modulation of immune response	[180]
	PEGDA hydrogel containing RGD and TMP	Magnetism	0.1 Hz 1T with periodic displacement	Diabetic wound healing	On-demand release of insulin for spatiotemporal glucose regulation	[195]
	pH/ROS dual response injectable glycopeptide hydrogel	pH/ROS	Schiff base and borate bonds	Diabetic wound healing	Hydrogel self-dissolving chains for spatiotemporal delivery of DS and MF	[205]
	HA hydrogel containing Zn^2+^ and Ga^3+^	pH	Humic acids	Infected bone defects	PH responds to release of Zn^2+^ and Ga^3+^ to eradicate bacterial infections and scavenge ROS	[207]
	HA hydrogels loaded with PDA nanoparticles	Redox	ROS degraded disulfide bonds	Diabetic wound healing	Size-dependent sequential release of functional molecules based on ROS response for spatiotemporal regulation of wound healing	[219]
	The hydrogel consisted of 8-arm-TK-NP, HS-PEG-SH, and RGD	Redox	ROS cleaved TK	Diabetic bone defects	Scavenged ROS and promoted the migration of BMSCs	[221]
	GelMA hydrogels containing PFC@PLGA/PPS NPs and CAT	Redox	ROS consumed by CAT	Bone defects	Demand-based oxygenation and scavenged ROS	[223]
	Hydrogel containing the complex PNAm-ICG-PTH microspheres	Light	808 nm 2 W cm^2^ 45 s	Bone defects	Photothermal effect promotes rapid release of early PTH	[183]
	Hydrogels containing ES, RF, and Fe_3_O_4_ NPs	Magnetism	1 Hz 6 % 30 min	Wound healing	Adjustment of the magnetic force and force/deformation frequency of the holder to produce mechanical stimulation	[194]
	Decellularized ECM/RSF hydrogels containing Fe_3_O_4_ NPs	Magnetism	6 % 120–130 mT 150 × 150 × 10 mm^3^	Bone defects	Mechanical stimulation occurred in response to SMF that evoked ECM/RSF/MNP	[198]
	HA hydrogels containing DMSA@Fe_3_O_4_ NPs	Magnetism	-2T to +2T 2 h	Spinal cord injury	Mechanical stimulation induces neuronal proliferation and differentiation	[203]
Nanoparticles	Platelet membrane-encapsulated PLGA nanoparticles containing BNN6	US	1 MHz 1W cm^2^ 5 min	Myocardial ischemia/reperfusion injury	The released of NO caused by US stimulated to rejuvenation of endothelial cells	[173]
	Ce6 combined with silica nanoparticles	Light	980 nm 1.0 W cm^2^ 60 s	THP-1 derived foam cells	Early and rapid clearance of ROS mitigates cellular autophagy	[185]
	MMS and Au NPs containing VEGF, aV, and PTX	Light	808 nm 2.5 W cm^2^ 10 min	Atherosclerosis	Thermophoresis penetrated into damaged blood vessels and rapidly releases vascular endothelial growth factor	[189]
	NPs composed of ZOL@PLGA NPs, SPIO and Yoda1	Magnetism	Round magnets (maximum strength = 6.6 G)	Osteoporosis bone defects	Aggregated at the site of injury and produced a mechanical stimulus	[199]
	Self-assembled dextran-containing shell nanoparticles PA/ASePSD	pH	Schiff base bonds	Atherosclerosis	High concentrations of ROS disrupt the diselenide bond and release astaxanthin, SS-31 peptide	[209]
	ECA-HP-β-CD NPs containing ISDN	Redox-	ROS cleaved the borate bond	Implants induced damage of endothelium	Consumption of ROS relieves endoplasmic reticulum stress	[226]
	Artificially aged erythrocyte-encapsulated PEI-Se Se-PCL-Se-Se-PEI NPs	Redox	Diselenide bonds	Spine cord injury	ROS-responsive degradation of micelles to release riluzole	[228]
Microspheres	GelMA/HepMA microspheres containing oxygen-carrying nanobubbles and BMP2.	US	650 kHz 1.5W cm^2^ 1min	Bone defects	US Stimulates Rapid Release of Oxygen to Improve Early Hypoxic Microenvironment	[169]
Microneedles	Microneedles containing VP and cross-linked heparin	Light	690 nm 25 mW cm^2^ 10min	Wound healing	Stimulated VP and released large amounts of ROS	[178]
	Glucose responsive bio-oxygenated microneedles	Redox	SDA and borate bonds in FPBAs	Diabetic wound healing	ROS trigger the decomposition of peracetic acid to produce oxygen and increase tissue oxygenation	[218]

## CRediT authorship contribution statement

**Xu He:** Writing – original draft, Visualization, Investigation. **Zeyu Han:** Writing – original draft, Methodology, Investigation. **Yunxuan Ruan:** Writing – original draft, Investigation. **Zijie Wang:** Visualization, Investigation. **Bo Liao:** Validation, Investigation. **Xinhe Li:** Validation, Software. **Jindong Tan:** Validation, Investigation. **Xiaoyu Han:** Writing – review & editing, Funding acquisition, Conceptualization. **Jieliang Shen:** Supervision, Resources, Conceptualization. **Dingqun Bai:** Writing – review & editing, Resources, Conceptualization.

## Declaration of competing interest

The authors declare that they have no known competing financial interests or personal relationships that could have appeared to influence the work reported in this paper.

## Data Availability

No data was used for the research described in the article.

## References

[1] Wynn T.A., Vannella K.M. (2016). Immunity.

[2] Newman H. (2021). Biomaterials.

[3] Eming S.A. (2017). Science.

[4] Han X. (2023). Biomaterials.

[5] Chi Z. (2024). Nature.

[6] Younesi F.S. (2024). Nat. Rev. Mol. Cell Biol..

[7] Gerlach B.D. (2021). Cell Metab..

[8] Liu S. (2023). Cell.

[9] Neves J. (2016). Science.

[10] Martino M.M. (2014). Science.

[11] Naba A. (2024). Nat. Rev. Mol. Cell Biol..

[12] Han X. (2023). Nano Today.

[13] Rodrigo-Navarro A. (2021). Nat. Rev. Mater..

[14] Mitrousis N. (2018). Nat. Rev. Mater..

[15] Christman K.L. (2019). Science.

[16] Pham S.H. (2020). Pharmaceutics.

[17] Lu Y. (2016). Nat. Rev. Mater..

[18] Xue L. (2024). Nat. Rev. Mater..

[19] Tapeinos C. (2022). Small.

[20] Zhang X. (2024). Adv. Healthcare Mater..

[21] Mura S. (2013). Nat. Mater..

[22] Hartgerink J.D. (2001). Science.

[23] Napoli A. (2004). Nat. Mater..

[24] Johnston R.J. (2019). Nature.

[25] Liu Y. (2020). Sci Immunol.

[26] Li X. (2023). Nat. Biomed. Eng..

[27] Nag S. (2024). Mater. Today Chem..

[28] Li X. (2024). Adv. Funct. Mater..

[29] Chagri S. (2022). Nat. Rev. Chem.

[30] Godwin J.W. (2017). NPJ Regen. Med..

[31] Ramírez-Valle F. (2024). Nat. Rev. Drug Discov..

[32] Singer A.J. (2021). Tissue Eng. B Rev..

[33] Peña O.A., Martin P. (2024). Nat. Rev. Mol. Cell Biol..

[34] Rodrigues M. (2019). Physiol. Rev..

[35] Han X. (2023). Mol. Pharm..

[36] Wang X. (2024). Adv. Healthcare Mater..

[37] Haensel D. (2020). Cell Rep..

[38] Aamar E. (2021). J. Invest. Dermatol..

[39] Mann K.G. (2009). Hämostaseologie.

[40] Heldin C.H., Westermark B. (1999). Physiol. Rev..

[41] Blobe G.C. (2000). N. Engl. J. Med..

[42] Furie B., Furie B.C. (2008). N. Engl. J. Med..

[43] DiPietro L.A. (1995). Am. J. Pathol..

[44] Wilkinson H.N., Hardman M.J. (2020). Open Biol.

[45] Yang J. (2024). Chem. Eng. J..

[46] Kimball A.S. (2017). Front. Immunol..

[47] Oliva N., Almquist B.D. (2020). Adv. Drug Deliv. Rev..

[48] Ansell D.M., Izeta A. (2015). Exp. Dermatol..

[49] Fu W. (2024). Chem. Eng. J..

[50] Caley M.P. (2015). Adv. Wound Care.

[51] Gurtner G.C. (2008). Nature.

[52] Einhorn T.A., Gerstenfeld L.C. (2015). Nat. Rev. Rheumatol..

[53] Saul D., Khosla S. (2022). Endocr. Rev..

[54] Claes L. (2012). Nat. Rev. Rheumatol..

[55] Hofbauer L.C. (2024). J. Clin. Investig..

[56] Zhou X. (2024). Adv. Mater..

[57] Chow S.K.-H. (2022). J. Orthop. Transl..

[58] Ono T., Takayanagi H. (2017). Curr. Osteoporos. Rep..

[59] Schlundt C. (2021). Acta Biomater..

[60] Gibon E. (2016). Stem Cell Res. Ther..

[61] Marsell R., Einhorn T.A. (2011). Injury.

[62] Loi F. (2016). Bone.

[63] Xiao L. (2022). Mater. Today.

[64] Wang H. (2023). Genes Dis..

[65] Schlundt C. (2023). Cells.

[66] Sivaraj K.K. (2020). eLife.

[67] Ruan Z. (2023). Bone Res..

[68] Phillips A.M. (2005). Injury.

[69] He X. (2023). Cell. Mol. Biol. Lett..

[70] Zhang M. (2024). Bioact. Mater..

[71] Saul D., Khosla S. (2022). Endocr. Rev..

[72] Wang T. (2023). Adv. Healthcare Mater..

[73] Weber C., Noels H. (2011). Nat. Med..

[74] Getz G.S., Reardon C.A. (2024). Int. J. Mol. Sci..

[75] Mao C. (2021). Aging (Albany NY).

[76] Weitz J.I., Fazio S. (2019). Circ. Res..

[77] Ma D. (2023). Chem. Eng. J..

[78] Libby P. (2021). Nature.

[79] Litwak K.N. (1998). Metabolism.

[80] Hernández-López P. (2024). J. Biomech..

[81] Falk E. (2006). J. Am. Coll. Cardiol..

[82] Li X. (2022). Comput. Methods Progr. Biomed..

[83] Lundberg J.O. (2015). Nat. Rev. Drug Discov..

[84] Yang T. (2018). Adv. Sci. (Weinh.).

[85] Qian S. (2020). Comput. Biol. Med..

[86] Schake M.A. (2023). iScience.

[87] Liu Y. (2024). Adv. Healthcare Mater..

[88] Jo-Watanabe A. (2024). Nat. Commun..

[89] Kampourakis T. (2024). Nat. Commun..

[90] Li J. (2023). Angew. Chem. Int. Ed..

[91] Hu X. (2023). Signal Transduct. Targeted Ther..

[92] Ahuja C.S. (2017). Nat. Rev. Dis. Primers.

[93] Zrzavy T. (2021). Brain.

[94] Nathan F.M., Li S. (2017). Neural Regen. Res..

[95] Li C. (2024). Bone Res..

[96] Li X. (2020). J. Neuroinflammation.

[97] Tremoleda J.L. (2016). Eur. J. Nucl. Med. Mol. Imag..

[98] Mu C. (2024). Mol. Imag. Biol..

[99] Silva N.A. (2014). Prog. Neurobiol..

[100] Hara M. (2017). Nat. Med..

[101] Lovering R. (1993). Proc. Natl. Acad. Sci. USA.

[102] Liu H. (2017). Neurochem. Res..

[103] Ren Z.X. (2023). Neural Regen. Res..

[104] Huang Y.Y. (2024). Biol. Psychiatry.

[105] Hampel H. (2021). Biol. Psychiatry.

[106] Bhatt S. (2020). Drug Discov. Today.

[107] Chaudhari V. (2023). Neurotox. Res..

[108] Iram F. (2024). Ageing Res. Rev..

[109] Wanionok N.E. (2024). Ageing Res. Rev..

[110] Nation D.A. (2019). Nat. Med..

[111] Liu H.-J., Xu P. (2022). Adv. Drug Deliv. Rev..

[112] Kim K.-T. (2018). Future Med. Chem..

[113] Popa-Wagner A. (2015). J. Neural Transm..

[114] Mason T.J., Sun D.-W. (2005). Emerging Technologies for Food Processing.

[115] Han W. (2024). Adv. Funct. Mater..

[116] Lorsung R.M. (2020). Front. Bioeng. Biotechnol..

[117] Wu Y. (2021). Nat. Biomed. Eng..

[118] Zamfirov L. (2024). Nat. Biomed. Eng..

[119] Carpentier A. (2016). Sci. Transl. Med..

[120] Wang J. (2019). Neurosurgery.

[121] Beachy S.H., Repasky E.A. (2011). Int. J. Hyperther..

[122] Sheybani N.D. (2020). Theranostics.

[123] Yamamoto T. (2024). Chem. Eng. Sci..

[124] Zheng Y. (2024). Adv. Mater..

[125] Tang Y. (2022). J. Mater. Chem. B.

[126] Sun W. (2019). Nano Res..

[127] Morishita K. (2015). Physiotherapy.

[128] Xu Z. (2023). J. Orthop. Res..

[129] Cui X. (2023). ACS Nano.

[130] Sun B. (2022). Biomaterials.

[131] Pierangeli D. (2023). Nat. Commun..

[132] Paolillo A.R. (2015). Laser Med. Sci..

[133] Lee H.P., Gaharwar A.K. (2020). Adv. Sci..

[134] Li L. (2019). Adv. Mater..

[135] Overchuk M. (2023). ACS Nano.

[136] Zhi D. (2020). J. Contr. Release.

[137] Khorsandi K. (2020). Curr. Stem Cell Res. Ther..

[138] Saleh M.S. (2024). Laser Med. Sci..

[139] Zhang X. (2022). Engineering.

[140] Pesqueira T. (2018). J. Cell. Physiol..

[141] Li Z. (2021). J. Contr. Release.

[142] Thévenot J. (2013). Chem. Soc. Rev..

[143] Lu C. (2021). Chem. Soc. Rev..

[144] Alphandéry E. (2017). Biomaterials.

[145] Peng H. (2017). Adv. Healthcare Mater..

[146] Materón E.M. (2021). Appl. Surf. Sci. Adv..

[147] Ding H. (2024). J. Mater. Chem. B.

[148] Hajjar S., Zhou X. (2023). Trends Immunol..

[149] Qiu Y. (2024). Int. J. Biol. Macromol..

[150] Zhu Y. (2020). Adv. Funct. Mater..

[151] Han Z. (2023). Nanoscale Horiz.

[152] Qu K. (2022). ChemPhysMater.

[153] Shinn J. (2022). J Pharm Investig.

[154] Kanamala M. (2016). Biomaterials.

[155] Kumar P. (2019). Macromol. Biosci..

[156] Castejon-Vega B. (2023). Antioxidants.

[157] Sies H. (2024). Nat. Rev. Mol. Cell Biol..

[158] Zhang J. (2024). Cell Metab..

[159] Muri J., Kopf M. (2021). Nat. Rev. Immunol..

[160] Abed H.F. (2022). Nanomaterials.

[161] Mollazadeh S. (2021). Mater. Sci. Eng. C.

[162] Husseini G.A. (2013). Colloids Surf. B Biointerfaces.

[163] Zhou Y. (2024). ACS Nano.

[164] Barman S.R. (2023). Sci. Adv..

[165] Wang X. (2025). Biomaterials.

[166] Han X. (2023). Adv. Funct. Mater..

[167] Choudhry H., Harris A.L. (2018). Cell Metab..

[168] Sheehy E.J. (2012). Biochem. Biophys. Res. Commun..

[169] Chen S. (2024). Adv. Funct. Mater..

[170] Liang W. (2024). Cell Metab..

[171] Jing H. (2018). Cell Death Dis..

[172] Zheng W. (2024). Biomaterials.

[173] Xu L. (2023). Small Struct..

[174] Kim T. (2023). Nat. Biomed. Eng..

[175] Koumura N. (1999). Nature.

[176] Ikeda T. (2003). Adv. Mater..

[177] Opdenakker G. (2018). Trends Immunol..

[178] Zhang Y. (2023). Nat. Commun..

[179] Hong Y. (2024).

[180] Wu M. (2024). Adv. Sci..

[181] Wein M.N., Kronenberg H.M. (2018). Cold Spring Harb. Perspect. Med..

[182] Dang M. (2017). Biomaterials.

[183] Kuang L. (2021). Adv. Funct. Mater..

[184] Ma C. (2024). Circ. Res..

[185] Han X.B. (2017). Cell Death Dis..

[186] Wang J. (2024). Nat. Commun..

[187] He M. (2018). Nano Energy.

[188] Cao Y. (2024). J. Am. Chem. Soc..

[189] Li X. (2021). ACS Appl. Mater. Interfaces.

[190] LaMer V.K., Dinegar R.H. (1950). J. Am. Chem. Soc..

[191] Gupta A.K., Gupta M. (2005). Biomaterials.

[192] Skalli O. (1986). J. Cell Biol..

[193] Serini G., Gabbiani G. (1999). Exp. Cell Res..

[194] Zhu M. (2024). Small.

[195] Shou Y. (2023). Adv. Mater..

[196] Paul G.R. (2018). Curr. Osteoporos. Rep..

[197] Nakamura H. (2013). J. Bone Miner. Res..

[198] Liang H.-F. (2023). Adv. Healthcare Mater..

[199] Guan H. (2024). Adv. Mater..

[200] Fan B. (2022). Bone Res.

[201] Holmes D. (2017). Nature.

[202] Yuan T. (2021). Adv. Mater..

[203] Zhang W. (2024). Adv. Sci..

[204] Kazunori K. (1993). J. Contr. Release.

[205] Wu Y. (2022). J. Contr. Release.

[206] Xu Z. (2024). Adv. Funct. Mater..

[207] Zha K. (2024). Adv. Sci..

[208] Mundi S. (2018). Cardiovasc. Res..

[209] Xu H. (2023). Adv. Mater..

[210] Woo Y.C. (2004). Anesthesiology.

[211] Kigerl K.A. (2009). J. Neurosci..

[212] David S., Kroner A. (2011). Nat. Rev. Neurosci..

[213] Xi K. (2020). Nat. Commun..

[214] Wei Y. (2025). Acta Pharm. Sin. B.

[215] Kwon G. (1994). J. Contr. Release.

[216] Rothenfluh D.A. (2008). Nat. Mater..

[217] Lei H., Fan D. (2022). Adv. Sci. (Weinh.).

[218] Liu H. (2024). Adv. Funct. Mater..

[219] Sun Z. (2024). Chem. Eng. J..

[220] Carlier A. (2015). J. Theor. Biol..

[221] Zhang Q. (2024). Small.

[222] Krinner A. (2009). Cell Prolif..

[223] Sun H. (2023). Bioact. Mater..

[224] Chen S. (2021). Bio-Design Manuf..

[225] Hu D. (2020). ACS Nano.

[226] Li J. (2024). Bioact. Mater..

[227] Milich L.M. (2019). Acta Neuropathol..

[228] Liu J. (2024). Adv. Funct. Mater..

[229] Wang J. (2024). Nat. Commun..

[230] Guo C. (2024). Regen. Biomater..

[231] Proksch E. (2018). J. Dermatol..

[232] Siah C.R. (2019). J. Clin. Nurs..

[233] Guan Y. (2021). Sci. Adv..

[234] Mascharak S. (2021). Science.

[235] Dhawan U.K. (2021). Pharmacol. Res..

[236] Asada Y. (2020). Pathol. Int..

